# Synthesis and evaluation of 3′-[^18^F]fluorothymidine-5′-squaryl as a bioisostere of 3′-[^18^F]fluorothymidine-5′-monophosphate†

**DOI:** 10.1039/d1ra00205h

**Published:** 2021-03-29

**Authors:** D. Brickute, A. Beckley, L. Allott, M. Braga, C. Barnes, K. J. Thorley, E. O. Aboagye

**Affiliations:** Comprehensive Cancer Imaging Centre, Faculty of Medicine, Imperial College London, Hammersmith Hospital W12 0NN London UK eric.aboagye@imperial.ac.uk; University of Kentucky, Department of Chemistry Lexington KY 40506 USA

## Abstract

The squaryl moiety has emerged as an important phosphate bioisostere with reportedly greater cell permeability. It has been used in the synthesis of several therapeutic drug molecules including nucleoside and nucleotide analogues but is yet to be evaluated in the context of positron emission tomography (PET) imaging. We have designed, synthesised and evaluated 3′-[^18^F]fluorothymidine-5′-squaryl ([^18^F]SqFLT) as a bioisostere to 3′-[^18^F]fluorothymidine-5′-monophosphate ([^18^F]FLTMP) for imaging thymidylate kinase (TMPK) activity. The overall radiochemical yield (RCY) was 6.7 ± 2.5% and radiochemical purity (RCP) was >90%. Biological evaluation *in vitro* showed low tracer uptake (<0.3% ID mg^−1^) but significantly discriminated between wildtype HCT116 and CRISPR/Cas9 generated TMPK knockdown HCT116^shTMPK−^. Evaluation of [^18^F]SqFLT in HCT116 and HCT116^shTMPK−^ xenograft mouse models showed statistically significant differences in tumour uptake, but lacked an effective tissue retention mechanism, making the radiotracer in its current form unsuitable for PET imaging of proliferation.

## Abstract 2

[^18^F]SqFLT was developed to bypass thymidine kinase 1 (TK1) and evaluated for PET imaging of DNA synthesis.

## Graphical abstract

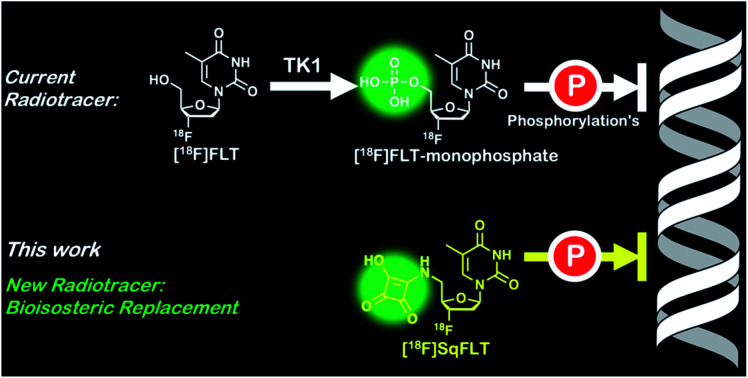

## Introduction

Bioisosteres improve the biological or physical properties of a molecule (*e.g.* toxicity, bioavailability, metabolism) by replacing problematic functional groups without making significant change to the structure.^1^ Problematic moieties can hinder the progression of otherwise promising lead candidates from entering clinical trials and therefore, their bioisosteric replacement is more cost-effective than implementing an alternative pharmacophore. Acidic functional groups are important in drug discovery with >450 marketed drugs containing a carboxylic acid; however, poor membrane permeability and metabolic instability support their bioisosteric replacement.^2^

The squaryl moiety, derived from squaric acid (3,4-dihydroxycyclobut-3-ene-1,2-dione), has been investigated as an acidic bioisostere in a number of compounds. The aromatic structure mimics the electrostatic properties of the phosphate group by forming resonance structures which result in two negatively charged carbonyls. The synthesis of novel oligodeoxynucleotide analogues containing the squaryl moiety as a phosphate mimic was reported;^3–6^ work by Sekine *et al.* has shown that base-recognition was maintained, opening up a potential for use in a variety of applications where precise hybridisation is required.^4^ Xie *et al.* (2003) developed protein tyrosine phosphatase (PTPase) inhibitors using the squaryl moiety as a non-hydrolysable bioisostere of phosphotyrosine with a reduced negative charge to improve bioavailability;^7^ squaric acid bound to the active site PTPase mimicked natural phosphate substrates. Potent squaryl containing analogues of γ-amino-butyric acid and l-glutamate were synthesised and investigated as neurochemically interesting molecules.^8^ Despite potential use in therapeutic molecules for many biological applications, squaryl (and phosphate bioisosteres more generally) has not been evaluated in the context of molecular imaging. An understanding of the relationship between squarate and phosphate in terms of biological properties (*i.e.* recognition by kinases), pharmacokinetics (PK) and metabolism may result in useful diagnostic imaging agents.

**Fig. 1:**
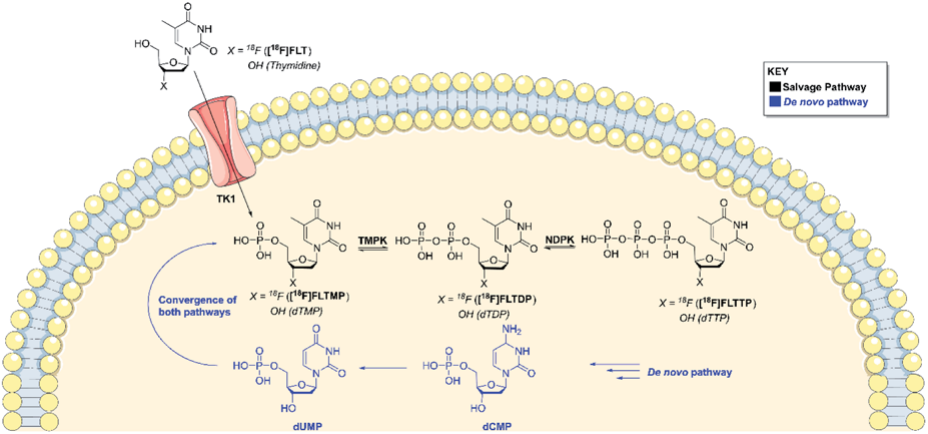
A schematic showing the *de novo* and salvage pathways of DNA synthesis. Both pathways converge at the formation of dTMP/[^18^F]FLTMP.

Positron emission tomography (PET) is a powerful and highly sensitive molecular imaging technique for probing living systems using sub-pharmacological doses of radiolabelled compounds for minimal biological perturbation.^9^ Phosphate groups play an important role in the uptake and retention of some PET radiopharmaceuticals inside the cell, including 3′-[^18^F]fluorothymidine ([^18^F]FLT), used to image rapidly proliferating cancer cells (Fig. 1). The *in vivo* metabolism of [^18^F]FLT is well understood.^10–12^ [^18^F]FLT follows the salvage pathway of DNA synthesis and undergoes three intracellular 5′-phosphorylations. Thymidine kinase-1 (TK1) converts [^18^F]FLT into [^18^F]FLT-monophosphate ([^18^F]FLTMP), which is subsequently phosphorylated into [^18^F]FLT-di/triphosphate ([^18^F]FLTDP/[^18^F]FLTTP) by thymidylate kinase (thymidine monophosphate kinase, TMPK; EC 2.7.4.9) and nucleotide diphosphate kinase (NDPK) respectively; preceding DNA incorporation. However, [^18^F]FLT uptake is largely dependent upon TK1 and therefore is unable to trace tumours that rely primarily on *de novo* thymidine monophosphate synthesis. We envisaged that imaging downstream of TK1, at the level of TMPK activity, where the *de novo* and salvage pathways converge would eliminate the dependence on TK1 activity. To achieve this, a radiotracer that mimics [^18^F]FLTMP would be required, as it is not a substrate for the TK1 enzyme but can be further phosphorylated by TMPK and NDPK. Despite [^18^F]FLTMP appearing to exhibit ideal characteristics for imaging TMPK activity, the radiosynthesis would be challenging and the radiotracer would be charged under physiological conditions (monophosphate p*K*_a_: 1.6, 6.6) preventing cell membrane permeability.^13,14^

Nucleotide derivatives with a masked monophosphate group (*i.e.* prodrugs) have several advantages over their unmasked counterparts such as overcoming reliance on nucleoside transporters for cellular uptake and phosphorylation by rate limiting kinases. The use of phosphoramidate pro-nucleotides (ProTides), which are clinically approved prodrugs exploited in nucleoside therapy, presents a potential solution.^15,16^ The ProTide moiety masks the charged phosphate for improved cellular membrane permeability yet releases the charged phosphate group once inside the cell. Cavaliere *et al.* (2020) developed a radiosynthetic route to access 3′-[^18^F]FLT ProTide; *in vitro* and *in vivo* biological evaluation is reportedly ongoing.^17^ The 3′-[^18^F]FLT ProTide is likely to passively diffuse across the cell membrane, however the PET readout may be rate-limited by the enzymatic cleavage of the ProTide moiety.

**Fig. 2:**
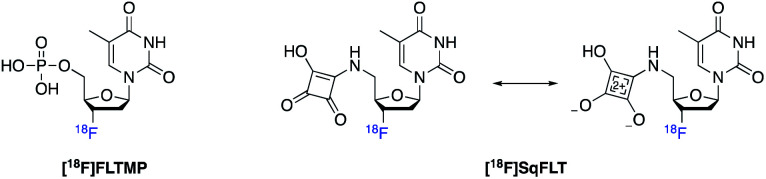
[^18^F]FLTMP and the proposed [^18^F]SqFLT phosphate mimic with resonance structure.

We proposed that the use a squaryl group as a phosphate bioisostere, substituted at the 5′-position of [^18^F]FLT, could be an alternative strategy. In order for a 5′-squaryl derivative of [^18^F]FLT to image proliferation downstream of TK1, it must not be transported into the cell by nucleoside transporters, *e.g.* equilibrative nucleoside transporter 1/2 (ENT1/2) and instead passively diffuse across the cell membrane. Experimental data on the membrane permeability of the squaryl group is limited; however, Lassalas *et al.* (2016) studied the structure activity relationship between organic acids and bioisosteres by experimentally determining and compiling data on lipophilicity (Log *D*_7.5_), acidity (p*K*_a_) and membrane permeability from parallel artificial membrane permeability assays (PAMPA).^2^ It was concluded that a simple squaryl substituted molecule may be significantly more cell membrane permeable than a similar phosphate despite comparable acidity and lipophilicity.^2^ This work was of particular interest for our application and encouraged the design, synthesis and biological evaluation of 3′-[^18^F]fluorothymidine-5′-squaryl ([^18^F]SqFLT) (Fig. 2). We hypothesised that the reduced negative charge and the potential increase in membrane permeability of the squaryl group compared to phosphate, may allow sufficient membrane permeability for PET imaging of proliferating cancer cells due to the high sensitivity of PET imaging.^2,7^ A high contrast PET image is not only dependent upon radiotracer uptake in target tissue, but rapid clearance from blood and non-target tissues (*e.g.* muscle).

The purpose of this study was two-fold: firstly, to evaluate if squaryl moiety was an effective bioisostere for the phosphate group in the context of a radiolabelled nucleoside ([^18^F]FLT); secondly, evaluate if [^18^F]SqFLT could image the DNA synthesis. This work represents the first radiosynthesis, *in vitro* and *in vivo* imaging and biodistribution of a radiolabelled squarylamide-thymidine derivative and may aid the development of therapeutic agents.

## Results and discussion

[^18^F]FLT has been studied extensively as a proliferation radiotracer and exhibits selective uptake *via* the thymidine salvage pathway, mediated by TK1. [^18^F]FLT is unable to image tumours that utilise the *de novo* pathway primarily, and therefore the development of a radiotracer that bypasses TK1 is clinically relevant.^18^ In this study, we proposed that [^18^F]SqFLT bearing a 5′-squaryl moiety as a phosphate bioisostere may result in a membrane permeable analogue of [^18^F]FLTMP. A requirement for successful PET imaging of TMPK activity is the phosphorylation of [^18^F]SqFLT, mimicking the formation of [^18^F]FLTDP, the diphosphate species of [^18^F]FLT. Molecular *in silico* modelling was used to determine if [^18^F]SqFLT could occupy the binding pocket of TMPK in a similar way to TMP and [^18^F]FLT; a first step towards phosphorylation if it acts as a substrate (as opposed to an enzyme antagonist).

### Molecular modelling

**Fig. 3:**
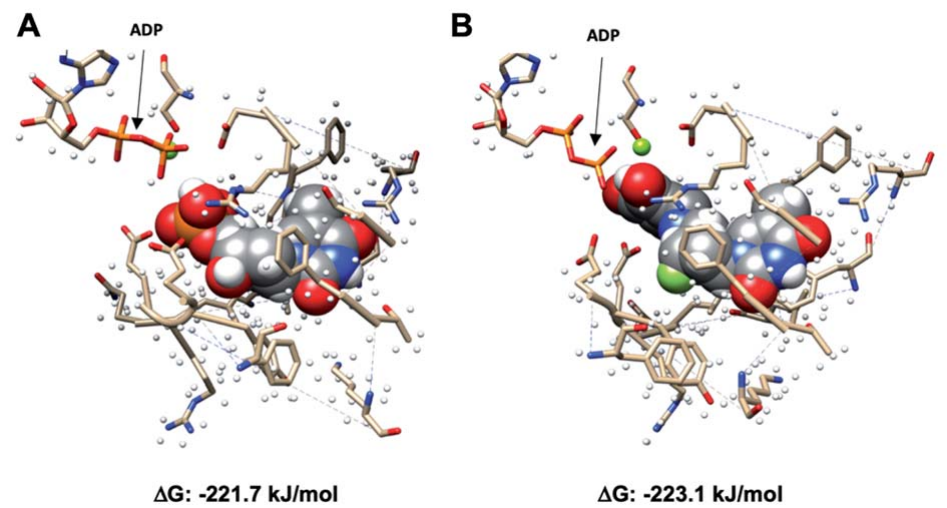
Molecular modeling of SqFLT and TMP (space-filled model) into the human ADP-bound dTYMK. (A) dTMP bound to the active site of TMPK (P-loop) in the presence of ADP and Mg^2+^; (B) SqFLT bound to the active site of TMPK (P-loop) in the presence of ADP and Mg^2+^; Δ*G* = free energies (kJ mol^−1^) of predicted binding interactions; color code: carbon: *black*; hydrogen: *white*; oxygen: *red*; nitrogen: *blue*; phosphorus: *orange*; fluorine: *large green*; magnesium: *small green*.

Using Gaussian 09 and UCSF Chimera,^19,20^ TMP with adenosine diphosphate (ADP), and SqFLT with ADP were docked into human TMPK to study the interactions between the ligands and the enzyme; the structures were geometrically optimized using the oniom method.^21^ The enzyme was treated using a molecular mechanics UFF force field, while the substrates (TMP and SqFLT) were modelled with density functional theory (B3LYP/6-31G*). Encouragingly, SqFLT occupied the catalytic site of TMPK, comparable to TMP (Fig. 3). Moreover, the hydroxyl group of the squaryl moiety was in close proximity to the phosphate of ADP, which could promote phosphotransfer in the presence of ADP and Mg^2+^. These preliminary data suggested a close relationship between SqFLT and bound TMP, by visual inspection of the image and the calculated free energy, encouraging further investigation.

### Chemical synthesis

**Scheme 1:**
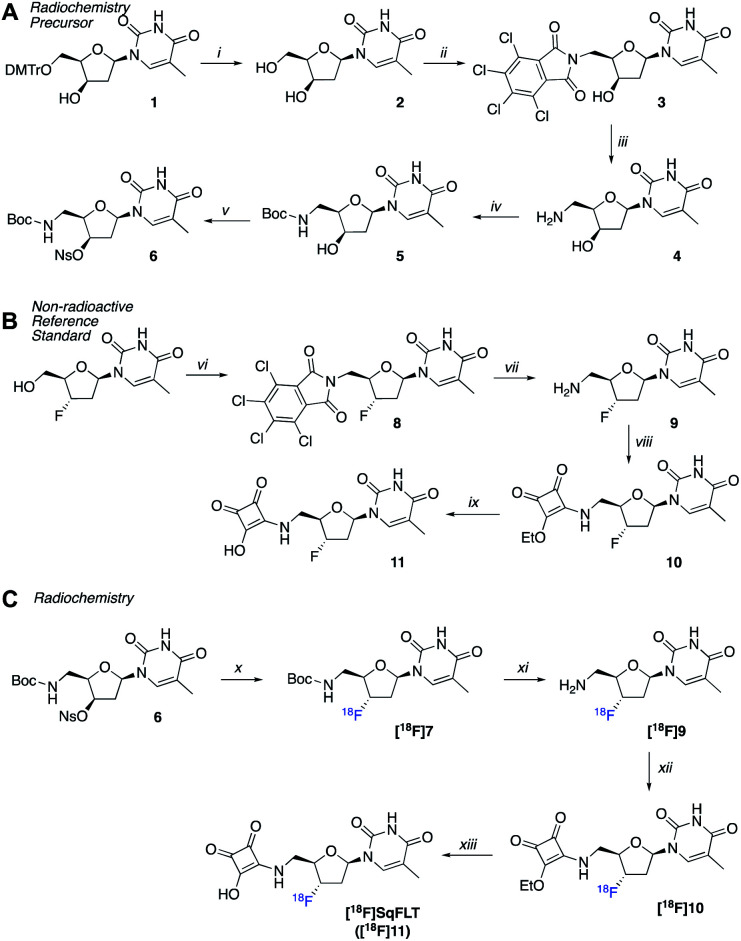
(A) Synthesis of precursor 6; (B) synthesis of reference compound 11; (C) radiosynthesis of [^18^F]SqFLT. *Reaction conditions*: (i) TFA, DCM, RT, 2 h; (ii) triphenylphosphine, tetrachlorophtalimide, DIAD, dry THF, RT, 4 days; (iii) ethylenediamine, MeCN, THF, EtOH, 60 °C, 1 h, then 45 °C, 2 h; (iv) Boc_2_O, NaHCO_3_, THF, H_2_O, 0 °C, 1 h, then RT, 2 h; (v) NsCl, pyridine, silver trifluoromethanesulfonate, RT, 2 h; (vi) triphenylphosphine, tetrachlorophtalimide, DIAD, dry THF, RT, 4 days; (vii) ethylenediamine, MeCN, THF, EtOH, RT, 2 h; (viii) 3,4-diethoxy-3-cyclobutene-1,2-dione, EtOH, RT, 1.5 h; (ix) HCl (4 M), acetone, RT, 16 h; (x) automated: [^18^F]TBAF, 2-methyl-2-butanol, MeCN, 125 °C, 30 min; (xi) phosphoric acid (2.29 M), 75 °C, 25 min; (xii) NH_3_ in MeOH, 3,4-diethoxy-3-cyclobutene-1,2-dione, pH 7, 50 °C, 40 min; (xiii) HCl (4N), EtOH, 85 °C, 40 min.

A radiosynthetic route to access [^18^F]SqFLT was established using precursor 6 (Scheme 1A). Compound 1 was synthesized following a literature procedure to ensure the 3′-OH was in the R-configuration so that the desired S-configuration was produced by inversion of stereochemistry upon nucleophilic substitution with [^18^F]fluoride (Scheme S1†). In brief, the 4,4′-dimethoxytrityl protecting group (DMTr) of 1 was removed from the 5′-OH moiety resulting in 2 in good yield (80%). The 5′-OH was converted into 5′-NH_2_ through a tetrachlorophthalic anhydride intermediate (3) giving compound 4 in a combined yield of 51%. The 5′-NH_2_ was boc-protected (5) and the nosylate leaving group installed on the 3′-OH resulting in radiochemical precursor 6. A non-radioactive standard for [^18^F]SqFLT ([^18^F]11) was synthesized, along with standards for key radiolabelled intermediates (Scheme 1B). Commercially available 3′-fluorothymidine ([^19^F]FLT) was modified at the 5′-OH position to bear a 5′-NH_2_ (9) as described above. The squaryl group was installed by reacting 3,4-diethoxy-3-cyclobutene-1,2-dione with the 5′-NH_2_ of 9 to produce 10 in a yield of 55%; subsequent hydrolysis of 10 produced [^19^F]SqFLT (11). All compounds were characterised by NMR and HRMS (spectra shown in the ESI, Fig. S1–S26†).

### Radiochemistry

Precursor 6 was radiolabelled using [^18^F]TBAF by S_N_2 displacement of the nosylate leaving group producing [^18^F]7. The nucleophilicity of the [^18^F]fluoride ion was modulated by the addition of 2-methyl-2-butanol in MeCN (1 : 1 v/v) as a polar and protic co-solvent.^22^ The absence of 2-methyl-2-butanol resulted in poor radiolabelling and the formation of unwanted radioactive products, suspected to be [^18^F]fluoronosylate. The ^18^F-fluorination of precursor 6 was automated using the GE FASTLab™ platform (Fig. S27†) and the resulting compound [^18^F]7 was purified by HLB-SPE in a 24.7 ± 7.4% RCY. Subsequent reactions were performed manually in Wheaton vials. The boc-group of [^18^F]7 was hydrolysed under acidic conditions to give [^18^F]9 and the reaction mixture was neutralized to pH 7. The free 5′-NH_2_ reacted with 3,4-diethoxy-3-cyclobutene-1,2-dione to give [^18^F]10 which was purified by semi-preparative radio-HPLC and formulated into EtOH for the final hydrolysis to [^18^F]SqFLT ([^18^F]11). Once neutralized, [^18^F]SqFLT was suitable for *in vitro* and *in vivo* evaluation. The overall radiochemical yield (RCY) was 6.7 ± 2.5% (decay corrected to start of synthesis) and the radiochemical purity (RCP) was >90%; the molar activity was 4.38 GBq μmol^−1^. The radiotracer was co-injected with [^19^F]SqFLT reference material which confirmed the identity of the product (Fig. S30†); the identity of intermediate product [^18^F]10 was also confirmed by co-injecting a reference standard (Fig. S29†). The overall 4 ± 1 h radiosynthesis was not fully optimized but produced [^18^F]SqFLT in sufficient quantity and quality for initial *in vitro* and *in vivo* evaluation. Attempts to simplify the radiochemistry by forming [^18^F]10 from a single ^18^F-fluorination reaction with an appropriate precursor proved unsuccessful and formed multiple radioactive products (data not shown).

The hydrophilicity of [^18^F]SqFLT (>90% RCP) was determined experimentally by its Log *D*_7.5_ using the shake flask method. The radiotracer was very hydrophilic with a Log *D*_7.5_ value of −2.90 ± 0.24 which was 2.4-fold lower than calculated Log *P* values for the tracer and [^18^F]FLTMP (Table S1†). The simple model squaryl compound (3-(benzylamino)-4-hydroxycyclobut-3-ene-1,2-dione) reported by Lassalas *et al.* (2016) exhibited significantly higher permeability compared to a phosphate analogue, however it was considerably less polar (Log *D*_7.4_ −1.4) than [^18^F]SqFLT, owed to the highly hydrophilic structure of nucleosides.^2^ Being a highly hydrophilic molecule, we were sceptical that [^18^F]SqFLT would be cell permeable and aimed to determine this experimentally in radioactive cell uptake experiments.

### *In vitro* evaluation

**Fig. 4:**
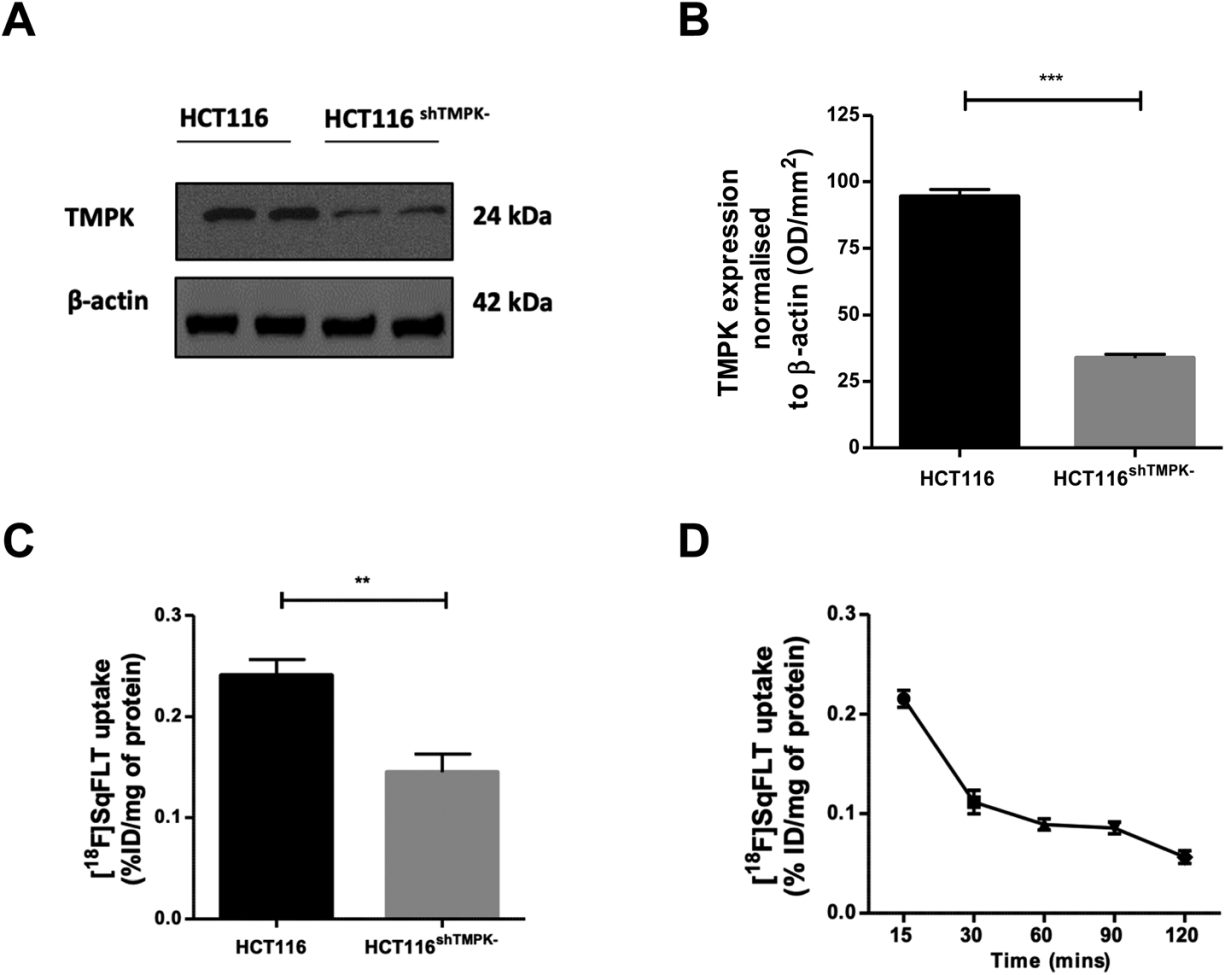
(A) Western blot analysis of TMPK expression in WT HCT116 and HCT116^shTMPK−^ cell lines; (B) quantification of TMPK expression normalized to β actin (*n* = 3; mean ± SEM); (C) uptake of [^18^F]SqFLT (0.74 MBq) in WT HCT116 and HCT116^shTMPK−^ cell lines following 60 min incubation (*n* = 6; mean ± SEM); (D) uptake of [^18^F]SqFLT (0.74 MBq) at 5 time points over 120 min (*n* = 3; mean ± SEM). Unpaired two-tailed *T*-test significance where *p* < 0.05 is indicated by **p <0.01 and ***p <0.001.

A CRISPR/Cas9 generated knockdown of TMPK in HCT116 cells (HCT116^shTMPK−^) was developed for use in radioactive uptake experiments alongside wildtype (WT) HCT116 cells, to determine if the uptake of [^18^F]SqFLT paralleled TMPK expression. Western blot analysis and quantification of HCT116^shTMPK−^ and WT HCT116 cell lines showed a 2.9-fold reduction of TMPK expression in the mutant cell line (33 ± 2%) (Fig. 4A and B) . The uptake of [^18^F]SqFLT in mutant HCT116^shTMPK−^ was significantly lower (0.14 ± 0.04% ID g^−1^) compared to WT HCT116 (0.24 ± 0.04% ID mg^−1^), despite low absolute uptake in both cell lines attributed to rate limiting permeability of [^18^F]SqFLT (Fig. 4C). The *in vitro* uptake kinetic profile was determined in WT HCT116 cells over 120 min (Fig. 4D). The peak uptake of [^18^F]SqFLT occurred between 0–15 min followed by a drop of 2-fold between 15–120 min; the efflux was not the result of an affinity of [^18^F]SqFLT to multidrug resistance proteins (Fig. S34†) and suggests that a permanently phosphorylated [^18^F]SqFLT species was not formed, which would have enhanced retention inside the cell. The uptake of [^18^F]SqFLT was confirmed to be independent of TK1 and nucleoside transporters (ENT1/2) by incubating pharmacological doses (1–10 μg mL^−1^) of [^19^F]SqFLT with [^18^F]FLT; thymidine was used as a positive control. An increase in thymidine concentration inversely correlated with [^18^F]FLT uptake in HCT116 cells, suggesting saturation of TK1 and ENT1/2; the same increase of [^19^F]SqFLT concentration did not inhibit [^18^F]FLT uptake (Fig. S33†). Encouraged by the *in vitro* uptake data, [^18^F]SqFLT was evaluated *in vivo*.

**Fig. 5:**
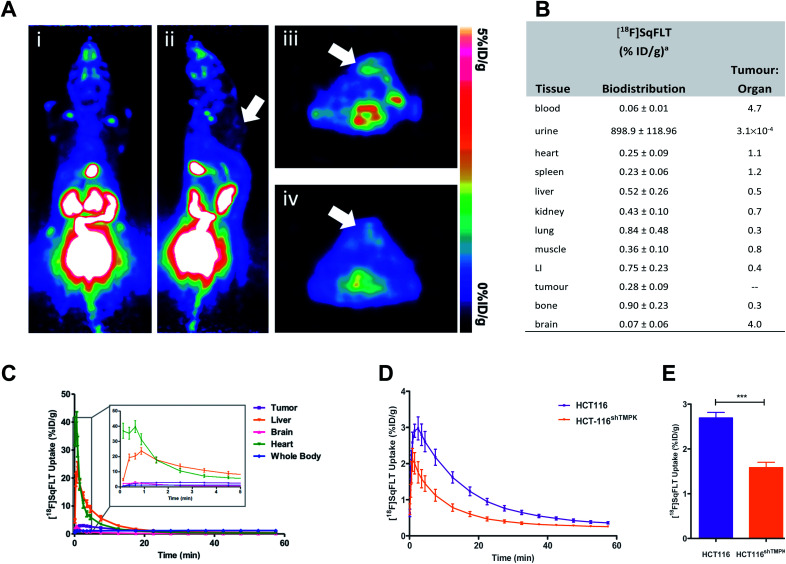
*In vivo* evaluation of [^18^F]SqFLT in female BALB/c athymic nude mice (6–8 weeks old) injected subcutaneously with either WT HCT116 or HCT116^shTMPK−^ cells (5 × 10^6^ cells per mouse). (A) Representative (i) coronal and (ii) sagittal PET-derived maximum intensity projection images of 60 min dynamic scans in athymic nude mice bearing HCT116 tumours (indicated by white arrow) injected with 0.74 MBq of [^18^F]SqFLT; (iii & iv) axial PET images of summed 60 min dynamic scans showing HCT116 tumours (indicated by white arrows). (B) Biodistribution analysis at 60 min p.i. (*n* = 5), LI = large intestine; (C) average time-activity curves (TACs) derived from region-of-interest analysis of PET imaging data (including detail of uptake kinetics between 0–5 min of [^18^F]SqFLT injection in HCT116 tumors, liver, brain, heart, whole body). Average TAC for bladder, kidneys and gallbladder are shown in the ESI (Fig. S35).† Data represent mean ± SEM (*n* >5) and are expressed as % of injected dose per gram (%ID g^−1^). (D) Time-activity curve of HCT116 and tumours derived from region-of-interest analysis of PET imaging data acquired over 60 min p.i.; (E) quantification of tumour uptake between 2–10 min post tracer injection.

### *In vivo* evaluation

The uptake of [^18^F]SqFLT was evaluated by dynamic PET imaging in female BALB/c athymic nude mice, used previously in our work with [^18^F]FLT-PET,^23–25^ inoculated with mutant HCT116^shTMPK−^ and WT HCT116 cells (Fig. 5A). The overall uptake into the tumours was low (Fig. 5B), however pharmacokinetic analysis (time–activity curve) of the 60 min scan showed significant differences between mutant and wildtype xenografts (Fig. 5D and E); peak radiotracer uptake occurred at 2–10 min post-injection, with 2.69 ± 0.12 *vs.* 1.58 ± 0.12% ID g^−1^ (WT and mutant, respectively). This suggests some specificity towards TMPK, but a lack of sensitivity to depict tumour proliferative activity. [^18^F]SqFLT was eliminated through the kidney, liver, and bladder (Fig. 5C); partial biliary elimination was inferred from the high radioactive accumulation in the gall bladder (Fig. S35†). Efflux of [^18^F]SqFLT through ABCB1 transporter was discounted as a mechanism for the rapid tumoral elimination by treating HCT116 cells with verapamil (3 μM), which did not change radiotracer uptake (Fig. S34†); this neither explains potential efflux by other ABC transporters nor nucleophilic attack on squaryl by cytoplasmic thiol containing compounds. Insights from the *in silico* modelling demonstrated that [^18^F]SqFLT may fit well into the binding pocket but unfortunately cannot prove [^18^F]SqFLT is a substrate for phosphorylation, or a selective antagonist. If [^18^F]SqFLT is a substrate for phosphorylation, to explain the *in vitro* and *in vivo* data, [^18^F]SqFLT-PO_4_ must degrade back to [^18^F]SqFLT and diffuses out of the cell. This hypothesis needs to be further tested, however there are limited conflicting reports on the use of squaric acid phosphates as DNA precursors.^3,4^ While we see selective uptake in paired TMPK-high and TMPK-low cells, the evidence from this work suggests that [^18^F]SqFLT behaves more in line with an antagonist than a substrate.^26–28^ Future studies of [^18^F]SqFLT or analogues should consider how the sex of a mouse cancer model influences tumour uptake and tissue biodistribution, as previously reported for [^18^F]FLT;^29^ given the poor *in vivo* performance of [^18^F]SqFLT, this was not further considered as part of this study. Incubation of [^18^F]SqFLT with human liver microsomes (HLM) showed no metabolism over 60 min.

## Conclusion

The aim of this study was to evaluate the use of the squaryl moiety as a bioisostere for the phosphate group in the context of fluorine-18 radiolabelled nucleosides. We synthesised [^18^F]SqFLT, a nucleoside derivative of [^18^F]FLTMP and investigated the radiotracer *in vitro* and *in vivo*. Promising *in silico* data showed that [^18^F]SqFLT may occupy the active site of TMPK in a favourable orientation for phosphorylation or antagonism of TMPK, which encouraged the synthesis and *in vitro* evaluation of radiotracer. Uptake was low, but statistically significant differences between wildtype and a CRISPR/Cas9 generated TMPK knockdown cell line was observed. The *in vivo* PET imaging of [^18^F]SqFLT in wildtype and TMPK mutant xenografts showed a similar statistically significant difference in uptake, but tumour washout was rapid as a result of poor cellular retention of the tracer. Low uptake *in vitro* and *in vivo* was likely due to poor passive diffusion across the cell membrane resulting from high hydrophilicity of tracer; further *in vitro* studies confirmed that [^18^F]SqFLT was not a substrate for nucleoside transporters TK1 or ENT1/2. Taken together, the data suggest that [^18^F]SqFLT is not an optimal radiotracer for imaging cellular proliferation, despite it's favourable properties for targeting TMPK activity.

This work presents synthetic and radiosynthetic routes to 5′-squaryl nucleosides that may be modified for other applications (*i.e.* synthesis of radiolabelled nucleoside dimers). In addition, the *in vivo* pharmacokinetics of squaryl-containing nucleosides are reported for the first time. We demonstrate the benefits and limitations of the squaryl group as a nucleoside phosphate bioisostere; while it may be considered a good mimic of the phosphate group in terms of physical properties (*i.e.* charge at physiological pH), it does not mimic the biological properties of the phosphate group and in this regard, it is a poor phosphate mimic.^3^ Further modulation of the acidity is required for [^18^F]SqFLT to cross the cell membrane (*i.e.* prodrug strategies). It is noteworthy that nucleoside derivatives are themselves hydrophilic (*c*Log *P* of [^18^F]FLT is −0.74) and therefore combining a charged squaryl moiety was detrimental to cell permeability in this context; this may not be the case when combined with lipophilic substrates as demonstrated in the literature.^2^

## Experimental

### Materials and methods

All reagents and solvents were purchased from commercial sources and were used without further purification unless otherwise stated. HPLC grade acetonitrile and trifluoroacetic acid (TFA), dimethyl sulfoxide (DMSO), dichloromethane (DCM), ethyl acetate (EtOAc), ethanol (EtOH) and hexane were purchased from Sigma Aldrich (Gillingham, Dorset, UK). [^18^F]Fluoride was produced by a GE PETrace cyclotron by 16 MeV irradiation of enriched [^18^O]H_2_O target, supplied by Alliance Medical Radiopharmacy Ltd (Warwick, UK). Automated radiosynthesis were performed using the GE FASTlab™ automated synthesis module (GE Healthcare Life Sciences, Amersham, UK). Solid phase extraction (SPE) cartridges were purchased from Waters and used according to the manufacturers recommended guidelines. Tetrabutylammonium hydrogen carbonate (TBAHC) 0.7 M in H_2_O was purchased from ABX GmbH (Radeberg, Germany). FLT was purchased from Carbosynth (Compton, UK). All RCY are decay-corrected to the start of synthesis, according to nomenclature guidelines.^30^ Radioactive semi-preparative HPLC was performed using a Shimadzu LC20-AT pump attached to a custom-built system, equipped with an Agilent Eclipse XDB-C18, 5 μm (250 × 9.4 mm) column. The mobile phase was 15% EtOH/85% H_2_O at a flow rate of 3 mL min^−1^. A detailed description of the analytical HPLC systems used in this work is presented in the ESI.† The radiosynthesis of [^18^F]FLT was performed on the GE FASTlab™ using commercially available synthesis cassettes (GE Healthcare, UK). Typically, [^18^F]fluoride (*ca.* 4 GBq, 2 mL), was used in the synthesis, which resulted in *ca* 440 MBq of [^18^F]FLT in >98% RCP and 10.9 ± 3.6% RCY.

### Molecular modelling

Gaussian 09 and UCSF Chimera were used to dock, TMP with adenosine diphosphate (ADP), and SqFLT with ADP into human TMPK.^20^ The structures were geometrically optimized using the oniom method. The enzyme was treated using a molecular mechanics UFF force field, while the substrates (TMP and SqFLT) were modelled with density functional theory (B3LYP/6-31G*).

### Chemical synthesis

#### 1-[5′-*O*-(4,4′-dimethoxytrityl)-2′-deoxy-β-d-lyxofuranosyl] thymine (1)^31^

A mixture of thymidine (1.5 g, 6.0 mmol) and dimethoxytrityl chloride (DMTrCl; 2.54 g, 7.5 mmol) in pyridine (30 mL) was stirred at RT for 16 h under N_2_. The solution was cooled to 0 °C and methanesulfonyl chloride (MsCl; 1.16 mL, 15.0 mmol) was added. After 3 h, ice water (1 mL) was added to the reaction mixture and allowed to stand for 1 h; it was then poured into ice water (500 mL) and stirred vigorously. The resulting precipitate was collected by suction filtration and dried under vacuum. The residue was dissolved in EtOH (120 mL) and NaOH (3 mL, 10 N) was added. After solution was heated for 1.5 h at 80 °C, neutralized with acetic acid and concentrated with toluene. The residue was purified by silica gel column chromatography (*n*-hexane:ethyl acetate, 1 : 3, v/v) to give 1 as a white solid (2.2 g, 65%). ^1^H NMR (400 MHz, DMSO-*d*_6_) *δ* 11.28 (s, 1H, NH), 7.60 (m, 1H), 7.47–7.38 (m, 2H), 7.35–7.18 (m, 7H), 6.95–6.81 (m, 4H), 6.11 (dd, *J* = 8.1, 2.3 Hz, 1H, H-1′), 5.20 (d, *J* = 3.5 Hz, 1H, 3′-OH), 4.24–4.05 (m, 2H, H-3′, H-4′), 3.73 (2× s, 6H, OCH_3_), 3.38 (dd, *J* = 10.4, 8.0 Hz, 1H, H-5′), 3.18 (dd, *J* = 10.3, 2.9 Hz, 1H, H-5′), 2.59–2.51 (m, 1H, H-2′), 1.86 (dd, *J* = 14.4, 2.5 Hz, 1H, H-2′), 1.64 (d, *J* = 1.1 Hz, 3H, 5-CH_3_). ^13^C NMR (101 MHz, DMSO-*d*_6_) *δ* 164.28 (CO-4), 158.49 (CHPh_3_), 150.97 (CO-2), 145.40 (CPh_3_), 137.25 (CH-6), 136.19 (CPh_3_), 135.98 (CPh_3_), 130.24 (CHPh_3_), 130.20 (CHPh_3_), 128.25 (CHPh_3_), 128.21 (CHPh_3_), 127.08 (CHPh_3_), 113.60 (CHPh_3_), 108.73 (C-5), 85.98 (CPh_3_), 84.63 (CH-4′), 83.81 (CH-1′), 69.42 (CH-3′), 63.29 (CH_2_-5′), 55.48 (CH_3_O), 41.28 (CH_2_-2′), 12.91 (5-CH_3_). HRMS (ESI) = 567.2110 (M + Na)^+^. Calc for C_31_H_32_N_2_O_7_Na: 567.2107.

#### 1-[2′-deoxy-β-d-lyxofuranosyl]thymine (2)

To a solution of 1 (1.57 g, 2.9 mmol) in DCM (30 mL) was added trifluoroacetic acid (TFA; 0.3 mL). The reaction mixture was stirred at RT for 2 h followed by concentration *in vacuo*. The residue was purified by silica gel column chromatography (DCM : MeOH, 97 : 3, v/v) to afford the product (560 mg, 80%) as a white solid. ^1^H NMR (400 MHz, DMSO-*d*_6_) *δ* 11.21 (s, 1H, NH), 7.80 (q, *J* = 1.1 Hz, 1H, H-6), 6.06 (dd, *J* = 8.4, 2.6 Hz, 1H, H-1′), 5.23 (d, *J* = 3.5 Hz, 1H, 3′-OH), 4.67 (t, *J* = 5.7 Hz, 1H, 5′-OH), 4.31–4.17 (m, 1H, H-3′), 3.88–3.55 (m, 3H, H-4′, H-5′, H-5′′), 2.62–2.50 (m, 1H, H-2′), 1.85 (ddd, *J* = 14.7, 2.7, 1.0 Hz, 1H, H-2′′), 1.76 (d, *J* = 1.2 Hz, 3H, 5-CH_3_). ^13^C NMR (101 MHz, DMSO-*d*_6_) *δ* 163.81 (CO-4), 150.56 (CO-2), 137.17 (CH-6), 108.64 (C-5), 84.77 (CH-4′), 83.44 (CH-1′), 68.64 (CH-3′), 59.57 (CH_2_-5′), 40.67 (CH_2_-2′), 12.46 (5-CH_3_). HRMS (ESI) = 243.0977 (M + H)^+^. Calc. for C_10_H_15_N_2_O_5_: 243.0981.

#### 1-[5-*N*-(4,5,6,7-tetrachloroisoindole-1,3-dione)5′-deoxy-2′-deoxy-β-d-lyxofuranosyl]thymine (3)

To a mixture of 2 (466 mg, 1.9 mmol), triphenylphosphine (TPP, 629 mg, 2.4 mmol) and tetrachlorophtalimide (TCP-NH, 683 mg, 2.4 mmol) anhydrous THF (50 mL) was added. The white slurry was stirred vigorously under N_2_ at RT. Diisopropyl azodicarboxylate (DIAD, 473 μL, 2.4 mmol) was added drop wise by a syringe and the reaction mixture, a yellow clear solution, was stirred at RT for 4 days. The resulting precipitate was collected by suction filtration and washed with THF to give the product (506 mg, 52%) as a white solid. ^1^H NMR (400 MHz, DMSO-*d*_6_) *δ* 11.24 (s, 1H, NH), 7.85 (m, 1H, H-6), 5.93 (dd, *J* = 8.0, 2.4 Hz, 1H, H-1′), 5.50 (d, *J* = 3.4 Hz, 1H, 3′-OH), 4.33 (q, *J* = 4.2 Hz, 1H, H-3′), 4.24–4.05 (m, 2H, H-4′, H-5′), 3.79 (dd, *J* = 14.1, 2.9 Hz, 1H, H-5′′), 2.57 (ddd, *J* = 14.6, 8.0, 5.2 Hz, 1H, H-2′), 1.97 (ddd, *J* = 14.5, 2.5, 1.1 Hz, 1H, H-2′′), 1.85 (d, *J* = 1.2 Hz, 3H, 5-CH_3_). ^13^C NMR (101 MHz, DMSO-*d*_6_) *δ* 163.84 (CO), 163.38 (CO), 150.39 (CO-2), 138.32 (CH-6), 136.84 (C), 128.17 (C), 128.13 (C), 108.33 (C-5), 84.23 (CH-4′), 80.45 (CH-1′), 68.60 (CH-3′), 40.81 (CH_2_-5′), 38.37 (CH_2_-2′), 12.49 (5-CH_3_). HRMS (ESI) = 505.9489 (M − H)^−^. Calc for C_18_H_12_N_3_O_6_Cl_4_: 505.9480.

#### 1-[5′-amino-5′-deoxy-2′-deoxy-β-d-lyxofuranosyl]thymine (4)

Synthesised by adapting a literature procedure.^32^ To a solution of compound 3 (270 mg, 0.53 mmol) in CH_3_CN/THF/EtOH (2 : 1 : 1 v/v, 40 mL) was added dropwise ethylenediamine (159 μL, 2.4 mmol). The reaction mixture was stirred first at 60 °C for 1 h, then at 45 °C for 2 h, after which it was concentrated *in vacuo*. The resulting material was purified by silica gel column chromatography (EtOAc : MeOH : TEA, 7 : 3 : 0.1) to provide the product (126 mg, 98%) as a pale yellow solid. ^1^H NMR (400 MHz, deuterium oxide) *δ* 7.79 (q, *J* = 1.2 Hz, 1H, H-6), 6.17 (dd, *J* = 8.4, 2.9 Hz, 1H, H-1′), 4.52 (ddd, *J* = 5.6, 3.3, 1.0 Hz, 1H, H-3′), 4.08 (td, *J* = 6.1, 3.3 Hz, 1H, H-4′), 3.17 (d, *J* = 6.1 Hz, 2H, H-5′, H-5′′), 2.78 (ddd, *J* = 15.4, 8.4, 5.6 Hz, 1H, H-2′), 2.14 (ddd, *J* = 15.4, 3.0, 1.0 Hz, 1H, H-2′′), 1.91 (d, *J* = 1.2 Hz, 3H, 5-CH_3_). ^13^C NMR (101 MHz, deuterium oxide) *δ* 168.67 (CO-4), 153.34 (CO-2), 138.19 (CH-6), 111.05 (C-5), 85.20 (CH-4′), 83.20 (CH-1′), 69.74 (CH-3′), 39.98 (CH_2_-5′), 39.33 (CH_2_-2′), 11.83 (5-CH_3_). HRMS (ESI) = 242.1140 (M + H)^+^. Calc. for C_10_H_16_N_3_O_4_: 242.1141.

#### 1-[5′-amino-5′-*N*-(-*t*-butoxycarbonyl-)-5′-deoxy-2′-deoxy-β-d-lyxofuranosyl]thymine (5)

To a solution of free amine 4 (88 mg, 0.37 mmol) in 1 : 1 mixture of THF/H_2_O (1.5 mL) NaHCO_3_ (93.3 mg, 1.11 mmol) and Boc_2_O (96.9 mg, 0.44 mmol) were added consecutively at 0 °C. After 1 h at 0 °C, the solution was stirred at RT for 2 h. After which time it was filtered and concentrated *in vacuo*. The residue was purified by silica gel column chromatography with 100% EtOAc to provide the product (80 mg, 64%) as a white solid. ^1^H NMR (400 MHz, DMSO-*d*_6_) *δ* 11.22 (s, 1H, NH), 7.79 (m, 1H, H-6), 6.89 (t, *J* = 6.0 Hz, 1H, NH-Boc), 6.03 (dd, *J* = 8.4, 2.4 Hz, 1H, H-1′), 5.26 (d, *J* = 3.3 Hz, 1H, 3′-OH), 4.17 (dt, *J* = 6.2, 3.2 Hz, 1H, H-3′), 3.79 (td, *J* = 6.4, 3.0 Hz, 1H, H-4′), 3.25 (t, *J* = 5.9 Hz, 2H, H-5′, H-5′′), 2.57 (ddd, *J* = 14.2, 8.5, 5.4 Hz, 1H, H-2′), 1.87 (dd, *J* = 14.8, 2.5 Hz, 1H, H-2′′), 1.77 (d, *J* = 1.2 Hz, 3H, 5-CH_3_), 1.38 (s, 9H, boc). ^13^C NMR (101 MHz, DMSO-*d*_6_) *δ* 163.81 (CO-4), 155.95 (CO-Boc), 150.53 (CO-2), 137.12 (CH-6), 108.68 (C-5), 83.53 (CH-4′), 82.49 (CH-1′), 77.97 (C-Boc), 68.55 (CH-3′), 40.31 (CH_2_-5′), 39.20 (CH_2_-2′), 28.21 (CH_3_-Boc), 12.44 (5-CH_3_). HRMS (ESI) = 342.1671 (M + H)^+^. Calc. for C_15_H_24_N_3_O_6_: 342.1665.

#### 1-[5′-amino-5′-*N*-(-*t*-butoxycarbonyl-)-5′-deoxy-2′-deoxy-3′-*O*-(4-nitrobenzenesulfonyl)-β-d-lyxofuranosyl]thymine (6)

The solid 5 (70 mg, 0.21 mmol) was dissolved in anhydrous pyridine (3 mL) at RT then the 4-nitrobenzenesulfonyl chloride (57.6 mg, 0.26 mmol) was added, followed by silver trifluoromethanesulfonate (66.8 mg, 0.26 mmol). The reaction mixture was stirred at 0 °C for 0.5 h and then RT for 2 h, after which time it was diluted with EtOAc (10 mL) and the precipitate (AgCl) that was formed, was filtered. The filtrate was extracted with brine (15 mL) and water (15 mL). The organic layer was dried with anhydrous Na_2_SO_4_ and concentrated *in vacuo*. The residue was purified by silica gel column chromatography with 100% EtOAc to give product (65 mg, 60%) as a yellow solid. ^1^H NMR (400 MHz, DMSO-*d*_6_) *δ* 11.26 (s, 1H, NH), 8.58–8.33 (m, 2H, H-Ph), 8.30–8.09 (m, 2H, H-Ph), 7.38 (m, 1H, H-6), 7.00 (t, *J* = 5.8 Hz, 1H, NH-Boc), 5.96 (dd, *J* = 7.7, 2.6 Hz, 1H, H-1′), 5.33 (dd, *J* = 4.9, 3.4 Hz, 1H, H-3′), 4.12 (dt, *J* = 8.0, 3.9 Hz, 1H, H-4′), 3.30–3.07 (m, 2H, H-5′, H-5′′), 2.78 (ddd, *J* = 15.7, 7.7, 5.1 Hz, 1H, H-2′), 2.32–2.16 (m, 1H, H-2′′), 1.72 (d, *J* = 1.2 Hz, 3H, 5-CH_3_), 1.36 (s, 9H, boc). ^13^C NMR (101 MHz, DMSO-*d*_6_) *δ* 163.59 (CO-4), 155.58 (CO-Boc), 150.76 (CO-2), 150.16 (C-Ph), 140.78 (C-Ph), 135.13(CH-6), 129.21 (CH-Ph), 124.99 (CH-Ph), 109.01 (C-5), 83.58 (CH-4′), 81.43 (CH-1′), 80.68 (CH-3′), 77.99 (C-Boc), 39.07 (CH_2_-5′), 38.40 (CH_2_-2′), 28.14 (CH_3_-Boc), 12.21 (5-CH_3_). HRMS (ESI) = 527.1461 (M + H)^+^. Calc. for C_21_H_27_N_4_O_10_S: 527.1448.

#### 5′-*N*-(4,5,6,7-tetrachloroisoindole-1,3-dione)-5′,3′-deoxy-3′-fluoro-thymidine (8)

To a mixture of alcohol (350 mg, 1.43 mmol), triphenylphosphine (TPP, 466 mg, 1.78 mmol) and tetrachlorophtalimide (TCP-NH, 507 mg, 1.78 mmol) anhydrous THF (20 mL) was added. The mixture, white slurry, was stirred vigorously under N2 at RT. Diisopropyl azodicarboxylate (DIAD, 350 μL, 1.78 mmol) was added by a syringe, and the reaction mixture, yellow clear solution, was stirred at RT for 4 days. The reaction mixture was concentrated *in vacuo*. The resulting precipitate was washed with acetone to give the product (585 mg, 80%) as a white solid. 1H NMR (400 MHz, DMSO) *δ* 11.34 (s, 1H, NH), 7.57 (d, *J* = 1.5 Hz, 1H, H-6), 6.14 (dd, *J* = 9.0, 5.7 Hz, 1H, H-1′), 5.58–5.12 (m, 1H, H-3′), 4.52–4.22 (m, 1H, H-4′), 4.07–3.78 (m, 2H, H-5′,H-5′′), 2.67–2.40 (m, 2H, H-2′, H-2′′), 1.82 (d, *J* = 1.2 Hz, 3H, 5-CH_3_). 19F NMR (376 MHz, DMSO-d6) *δ* −175.21.

^13^C NMR (101 MHz, DMSO) *δ* 163.61 (CO), 163.47 (CO), 150.33 (CO-2), 138.24 (TCP-C), 136.44 (CH-6), 128.26 (TCP-C), 128.13 (TCP-C), 109.68 (C-5), 94.04 (d, *J* = 176.1 Hz, CH-3′), 84.60 (CH-1′), 80.76 (d, *J* = 25.5 Hz, CH-4′), 40.21 (d, *J* = 10.3 Hz, CH_2_-5′), 35.77 (d, *J* = 20.2 Hz, CH_2_-2′), 11.89 (5-CH_3_). HRMS (ESI) = 509.9588 (M + H)^+^. Calc. for C18H13N3O5CI4F: 509.9593.

#### 5′-amino-5′,3′-deoxy-3′-fluoro-thymidine (9)

Synthesised by adapting a literature procedure.^32^ To a solution of compound 8 (500 mg, 0.98 mmol) in CH_3_CN/THF/EtOH (2 : 1 : 1, 20 mL) was added dropwise ethylenediamine (293 μL, 4.4 mmol). The reaction mixture was stirred at RT for 2 h, after which time it was concentrated *in vacuo*. The resulting material was purified by silica gel column chromatography (EtOAc : MeOH : TEA, 7 : 3 : 1%) to provide the product (158 mg, 66%) as a white solid. ^1^H NMR (400 MHz, DMSO-*d*_6_) *δ* 7.70 (q, *J* = 1.3 Hz, 1H, H-6), 6.16 (dd, *J* = 8.3, 6.6 Hz, 1H, H-1′), 5.43–5.20 (m, 1H, H-3′), 4.01 (dt, *J* = 28.3, 5.2 Hz, 1H, H-4′), 3.01–2.57 (m, 2H, H-5′, H-5′′), 2.46–2.25 (m, 2H, H-2′, H-2′′), 1.79 (d, *J* = 1.2 Hz, 3H, CH_3_-5). ^19^F NMR (376 MHz, DMSO-*d*_6_) *δ* −173.92. ^13^C NMR (101 MHz, DMSO-*d*_6_) *δ* 163.64 (CO-4), 150.48 (CO-2), 136.03 (CH-6), 109.78 (C-5), 94.67 (d, *J* = 173.6 Hz, CH-3′), 85.54 (d, *J* = 22.5 Hz, CH-4′), 83.61 (CH-1′), 42.96 (d, *J* = 10.0 Hz, CH_2_-5′), 36.18 (d, *J* = 20.3 Hz, CH_2_-2′), 12.13 (CH_3_-5). HRMS (ESI) = 244.1097 (M + H)^+^. Calc. for C_10_H_15_N_3_O_3_F: 244.1097.

#### 5′-amino-5′-*N*-(2-ethoxy-3,4-dioxocyclobuten-1-yl)-5′,3′-deoxy-3′-fluoro-thymidine (10)

Amine 9 (140 mg, 0.58 mmol) was dissolved in anhydrous EtOH (10 mL) and 3,4-diethoxy-3-cyclobutene-1,2-dione (94 μL, 0.64 mmol) was added. The resulting solution was stirred at RT for 1.5 h. After which time it was concentrated *in vacuo*. The resulting material was purified by silica gel column chromatography (DCM : MeOH, 95 : 5) to provide the product (116 mg, 55%) as a white solid. ^1^H NMR (400 MHz, DMSO-*d*_6_) presence of rotamers is observed: *δ* 11.38 (s, 1H, NH-3), 8.93 (s, 0.5H, NH), 8.75 (s, 0.5H, NH), 7.40 (m, 1H, H-6), 6.16 (q, *J* = 7.0 Hz, 1H, H-1′), 5.61–5.02 (m, 1H, H-3′), 4.65 (2× q, 2H, sqCH_2_), 4.27–4.01 (m, 1H, H-4′), 3.93–3.55 (m, 2H, H-5′, H-5′′), 2.49–2.20 (m, 2H, H-2′, H-2′′), 1.77 (, 3H, CH_3_-5), 1.35 (2× t, 3H, 2× sqCH_3_). ^19^F NMR (376 MHz, DMSO-*d*_6_) *δ* −175.01 and −175.50. ^13^C NMR (101 MHz, DMSO-*d*_6_) *δ* 189.26, 188.93 (Sq-C), 182.56, 182.26 (Sq-C), 177.42, 176.95 (Sq-C), 173.17, 172.41 (Sq-C), 163.56 (CO-4), 150.37(CO-2), 136.05, 135.90 (CH-6), 109.89 (C-5), 93.47 (d, *J* = 175.7 Hz, CH-3′), 84.06 (CH-1′), 82.65 (m, CH-4′), 68.94 (CH_2_-sq), 44.85 (m, CH_2_-5′), 35.57 (m, CH_2_-2′), 15.56 (CH_3_-sq), 12.04, 11.98 (CH_3_-5). HRMS (ESI) = 368.1249 (M + H)^+^. Calc. for C_16_H_19_N_3_O_6_F: 368.1258.

#### 5′-amino-5′-*N*-(2-hydroxy-3,4-dioxocyclobuten-1-yl)-5′,3′-deoxy-3′-fluoro-thymidine (11)

To a solution of ester (60 mg, 0.163 mmol) in acetone (3 mL) was added 4 M HCl (3 mL), and the solution was left to stir at RT overnight. The solvent was removed to dryness under vacuo to give a final compound as white solid (54 mg, 98%). ^1^H NMR (400 MHz, DMSO-*d*_6_) *δ* 11.38 (s, 1H, NH), 8.47 (t, *J* = 6.4 Hz, 1H, NH), 7.40 (q, *J* = 1.1 Hz, 1H, H-6), 6.16 (dd, *J* = 8.7, 6.0 Hz, 1H, H-1′), 5.32 (ddd, *J* = 54.1, 4.0, 2.0 Hz, 1H, H-3′), 4.19 (dtd, *J* = 27.0, 6.1, 5.6, 1.6 Hz, 1H, H-4′), 3.88–3.50 (m, 2H, 2H, H-5′, H-5′′), 2.48–2.29 (m, 2H, 2H, H-2′, H-2′′), 1.77 (d, *J* = 1.2 Hz, 3H, CH_3_-5). ^13^C NMR (101 MHz, DMSO-*d*_6_) *δ* 185.23 (Sq-C), 174.19 (Sq-C), 163.58 (CO-4), 150.39 (CO-2), 135.76 (CH-6), 109.96 (C-5), 93.72 (d, *J* = 175.7 Hz, CH-3′), 84.02 (CH-1′), 82.84 (d, *J* = 24.8 Hz, CH-4′) 44.78 (d, *J* = 9.8 Hz, CH_2_-5′), 35.75 (d, *J* = 20.3 Hz, CH_2_-2′), 12.01 (CH_3_-5).HRMS (ESI) = 338.0790 (M − H)^−^. Calc. for C_14_H_13_N_3_O_6_F: 338.0788.

#### Radiochemistry

The fluoride drying and first radiolabelling step to access [^18^F]9 was automated using the GE FASTlab™ with a custom designed cassette (Scheme S23†). Typically, [^18^F]fluoride (6 ± 2 GBq, 2 mL) target water trapped on a QMA-carbonate Sep-Pak SPE cartridge and eluted into the reactor using 800 μL of eluent solution (160 μL of TBAHC, 640 μL anhydrous MeCN). The eluate was evaporated to dryness under a stream of nitrogen at 120 °C for 12 min. After drying, precursor 6 (12 mg in 1.2 mL of 2-methyl-2-butanol/MeCN, 1 : 1 v/v) was added to the reactor and heated at 125 °C for 30 min. Once cooled, the reaction mixture was diluted in water (40 mL) and passed through an Oasis Plus HLB cartridge [pre-conditioned with EtOH (5 mL) and water (10 mL)]. Compound [^18^F]7 was retained on the cartridge and eluted manually into a clean Wheaton vial with EtOH (500 μL). Phosphoric acid (1 mL, 2.29 M) was added and the reaction was heated at 75 °C for 25 min to hydrolyse [^18^F]7 into [^18^F]9. Once cooled, the reaction mixture was neutralized by the addition of NH_3_ in MeOH (350 μL), followed by the addition of 3,4-diethoxy-3-cyclobutene-1,2-dione (15 μL) to give [^18^F]10. The resulting mixture was heated to 50 °C for 40 min and diluted into water (10 mL) for semi-preparative radio-HPLC purification. [^18^F]10 was collected into water (10 mL) and passed through an Oasis (30 mg) HLB cartridge [pre-conditioned with EtOH (5 mL) and water (10 mL)]. The cartridge was eluted in a minimum volume of EtOH (300 μL) into a clean Wheaton vial containing HCl (4 M, 200 μL). The reaction mixture was heated to 85 °C for 40 min, cooled and neutralized using NaOH (4 M) to give [^18^F]SqFLT (59.3 ± 15.6 MBq) for further evaluation.

#### Log *D*_7.5_ determination

To a 1.5 mL microcentrifuge tube was added PBS pH 7.5 (500 μL) and octanol (500 μL). The tube was shaken for 15 min, followed by centrifugation (13 000*g*, 5 min). [^18^F]SqFLT (3 MBq) in PBS (<5% EtOH) was added and the samples shaken for 30 min, followed by centrifugation (13 000*g*, 5 min). Aliquots from the octanol layer and PBS layer were removed (100 μL) and placed in counting tubes. Radioactivity was measured using a γ-counter and the partition coefficient was calculated using Log *D*_7.5_ = log_10_[Oct/PBS]. The Log *D*_7.5_ value was reported as mean ± SD (*n* = 3, triplicate analysis).

#### Cell culture

HCT116 (human colon cancer; LGC Standards, Teddington, Middlesex, UK) and subsequent knockdown HCT116^shTMPK−^ cells were cultured in Roswell Park Memorial Institute Medium 1640 supplemented with 10% foetal bovine serum, 2% Penicillin-Streptomycin (5000 U mL^−1^) and 1% l-Glutamine. Cells were maintained in a 5% CO_2_ humidified incubator at 37 °C and grown in 6-well plates at 300 000 cells per well or 1 500 000 cells (per 15 cm petri dish for HPLC) for the stated cell lines. All cells were grown for 24 h in a volume of 2 mL per well or 10 mL per petri dish unless stated otherwise. All cells were maintained at 60–70% confluence prior to experiments, passaged in our laboratory for fewer than 6 months on receipt and were tested mycoplasma free. Experiments were performed using cells under 10 passages.

#### Generating a CRISPR/Cas9 knockdown of TMPK

Guide RNA (gRNA) sequences for CRISPR/Cas9 were generated from CRISPR design web site (http://crispr.mit.edu/), provided by the Feng Zhang Lab.^33^ HCT116 cells were co-nucleofected with dTYMK CRISPR gRNAs 25926363 (GCGCGGGGCTCTCATAGTGC) or 25926373 (GCCACCGCGCCGAACTGCTC), and *Streptococcus pyogenes* Engen Cas9NLS (SpCas9) programmed with tracrRNA to produce a Cas9 Ribonucleoprotein mix.^33,34^ The two TMPK gRNAs target the exon 1 of the TMPK gene. Complementary oligonucleotides to gRNAs of interest were annealed and cloned into SpC CRISPR/Cas9-Puro vector (Addgene, Cambridge, MA). After two days, following transfection, cells were treated with 1 μg mL^−1^ of puromycin for three days. Two weeks later, colonies were isolated with the cloning cylinders. Clones were then prepared for DNA sequencing along with western blotting.

#### *In vitro* cell uptake

[^18^F]SqFLT (0.74 mBq per well) was added to individual wells and cells were incubated at 37 °C in a humidified atmosphere of 5% CO_2_ for 15, 30, 60, 90 and 120 min. Following incubation, cells were washed three times with 1× PBS and lysed once ice for 15 min using RIPA buffer (1 mL per well). Cell lysates were homogenized and transferred to radioactivity counting tubes. Radioactivity in each sample was counted using an auto-gamma counter (Perkin Elmer, London, UK). Total protein per sample was quantified using the Pierce™ BCA protein assay method. Decay corrected counts were normalized to protein concentration as percentage incubated dose per milligram of cellular protein (%ID per mg protein) in each sample.

#### *In vitro* competition assay between [^18^F]FLT and thymidine/[^19^F]SqFLT

HCT116 were plated and maintained at 60–70% confluence in complete media conditions as previously described. Following 24 h, cell media was aspirated and replaced with media containing either thymidine or [^19^F]SqFLT at log concentrations of 10^−3^–10^1^ μg mL^−1^ in a volume of 2 mL per well. Treated cells were incubated at 37 °C for 20 min before [^18^F]FLT was added to each well to yield a final concentration of 0.74 mBq per well. Cells were subsequently incubated in a humidified atmosphere of 5% CO_2_ for 1 h before being lysed and gamma counted as previously described.

#### Efflux assay

HCT116 cells were plated and maintained at 60–70% confluence in complete media conditions as previously described. Following 24 h, cell media was aspirated and treated with media containing 3 μM verapamil for 1 h prior to co-incubation with 0.74 mBq of [^18^F]SqFLT. Cells were subsequently incubated for a further 1 h before being lysed and gamma counted as previously described.

#### *In vitro* metabolite analysis

A published method for performing *in vitro* metabolite analysis using liver microsomes was adapted.^35^ [^18^F]SqFLT (40 μL, 3.5 MBq) was added to human liver microsomes (50 μL, 1 mg mL^−1^), NADPH regeneration system A (50 μL), NADPH regeneration system B (10 μL) and PBS (400 μL, 0.1 M) in a 1.5 mL microcentrifuge tube. The mixture was gently vortexed and incubated in aerobic conditions at 37 °C with gentle shaking for either 30 min or 60 min using a thermomixer. The mixture was transferred into a 15 mL plastic centrifuge tube and proteins were precipitated with ice-cold MeCN + 0.1% TFA (2 mL) and vortexed. Proteins were pelleted by centrifugation (12 000*g*, 3 min), and the supernatant removed and filtered (0.2 μm). The supernatant was diluted (1 : 9) with H_2_O + 0.1% TFA and injected onto a HPLC instrument bearing a 1 mL injection loop and PosiRam (LabLogic, Sheffield, UK) metabolite detector. Samples of a known volume were taken from the supernatant and the entire protein pellet were counted using an auto-gamma counter (Perkin Elmer, London, UK) to determine the extraction efficiency. All experiments were performed in triplicate (*n* = 3).

#### Western blotting

Cells were lysed in radioimmunoprecipitation assay (RIPA) buffer containing protease and phosphatase inhibitors (all from Sigma-Aldrich). Equal amounts of protein (20 μg) were resolved on 4–15% mini-protean TGX gels and transferred to PVDF membranes. Membranes were subsequently blocked for 1 h in 5% non-fat dry milk in phosphate buffered saline containing 0.1% v/v tween-20 (PBST) and incubated with β-Actin (Abcam, ab6276) and dTYMK Abcam (Abcam, ab15486) overnight at 4 °C. Following incubation, membranes were washed 3 × 15 min in PBST. Secondary HRP-conjugated mouse (Santa Cruz Biotechnology, sc-2004) and rabbit antibodies (Santa Cruz Biotechnology, sc-2005) were added to membranes and incubated for 1 h at room temperature. Signals were visualised using Amersham ECL Western Blotting Detection Reagent and Amersham Hyperfilm.

#### Biodistribution

Sample collection blood was collected by cardiac puncture into heparinized syringes and counted. Whilst under terminal anaesthesia, tissues of interest including heart, lung, liver, spleen, kidneys, muscle, bone, large intestines were collected in pre-weighed counting tubes. Radioactivity within tissue samples was counted using an auto-gamma counter (Perkin Elmer, London, UK), and then weighed to determine the mass of the tissue. Counts-per-minute (CPM) for each tissue sample was normalized to the total injected dose (0.74 mBq) of radioactivity to the animal to give %ID (injected dose), and then normalized to the weight of the counted tissue to give the radioactivity uptake as %ID g^−1^.

#### Animal tumour model

Female BALB/c athymic nude mice (6–8 weeks old), injected subcutaneously with either mutant HCT116^shTMPK−^ or WT HCT116 (Charles River UK Ltd., Margate, UK) at 5 × 10^6^ cells per mouse, using dynamic PET imaging (Fig. 5A). Inoculations were performed under 2% isoflurane/O_2_ anesthesia 7 days after animal arrival. All mice were aged 10–16 weeks with similar weight (20 ± 2 g) and kept under standard conditions in individually ventilated cages (maximum of 6 animals per cage) prior to experiments.

#### PET imaging

BALB/c mice (Charles River UK Ltd, Margate, UK) were anesthetized and scanned on a dedicated small animal PET scanner (G4 Genesis, Sofie Biosciences, Culver City, CA, USA) following a bolus injection of 0.75 MBq of [^18^F]SqFLT *via* a lateral tail vein cannula. Imaging was performed under 2% isoflurane/O_2_ anaesthesia. After tracer injection, emission scans were acquired in list-mode format (over 0–60 min – dynamic scans) to give decay-corrected values of radioactivity accumulation in tissues. The collected data were reconstructed with a 3-dimensional maximum likelihood estimation method 3D ML-EM (Sofie Biosciences). Cumulative images of the data were used for visualization of radiotracer uptake and to define tissue volumes of interest (VOIs) using Siemens Inveon Research Workplace software (Siemens Molecular Imaging, Inc. Knoxville, USA). Tissue radioactivity uptake values were normalized to injected dose and mouse weight.

#### Ethics statement

All animal experiments were done by licensed investigators in accordance with the UK Home Office Guidance on the Operation of the Animal (Scientific Procedures) Act 1986 (HMSO, London, UK, 1990) and within guidelines set out by the UK National Cancer Research Institute Committee on Welfare of Animals in Cancer Research.^36^ To limit the use of animals in this study, only four non-tumour bearing mice were used for PET imaging and metabolite analysis.

## Conflicts of interest

There are no conflicts to declare.

## Supplementary Material

RA-011-D1RA00205H-s001

## References

[cit1] Meanwell N. A. (2014). Top. Med. Chem..

[cit2] Lassalas P., Gay B., Lasfargeas C., James M. J., Tran V., Vijayendran K. G., Brunden K. R., Kozlowski M. C., Thomas C. J., Smith A. B., Huryn D. M., Ballatore C. (2016). J. Med. Chem..

[cit3] Dürr E. M., Doherty W., Lee S. Y., El-Sagheer A. H., Shivalingam A., McHugh P. J., Brown T., McGouran J. F. (2018). ChemistrySelect.

[cit4] Sato K., Seio K., Sekine M. (2002). J. Am. Chem. Soc..

[cit5] Seio K., Miyashita T., Sato K., Sekine M. (2005). Eur. J. Org. Chem..

[cit6] Lu M., Bin Lu Q., Honek J. F. (2017). Bioorg. Med. Chem. Lett..

[cit7] Xie J., Comeau A. B., Seto C. T. (2004). Org. Lett..

[cit8] Chan P. C. M., Roon R. J., Koerner J. F., Taylor N. J., Honek J. F. (1995). J. Med. Chem..

[cit9] Allott L., Aboagye E. O. (2020). Mol. Pharm..

[cit10] Barthel H., Cleij M. C., Collingridge D. R., Hutchinson O. C., Osman S., He Q., Luthra S. K., Brady F., Price P. M., Aboagye E. O. (2003). Cancer Res..

[cit11] Shields A. F., Grierson J. R., Muzik O., Stayanoff J. C., Lawhorn-Crews J. M., Obradovich J. E., Mangner T. J. (2002). Mol. Imaging Biol..

[cit12] Kenny L. M., Vigushin D. M., Al-Nahhas A., Osman S., Luthra S. K., Shousha S., Coombes R. C., Aboagye E. O. (2005). Cancer Res..

[cit13] Vanheusden V., Munier-Lehmann H., Pochet S., Herdewijn P., Van Calenbergh S. (2002). Bioorg. Med. Chem. Lett..

[cit14] Roy B., Depaix A., Périgaud C., Peyrottes S. (2016). Chem. Rev..

[cit15] Mehellou Y., Rattan H. S., Balzarini J. (2018). J. Med. Chem..

[cit16] McGuigan C., Pathirana R. N., Mahmood N., Devine K. G., Hay A. J. (1992). Antiviral Res..

[cit17] Cavaliere A., Probst K. C., Paisey S. J., Marshall C., Dheere A. K. H., Aigbirhio F., McGuigan C., Westwell A. D. (2020). Molecules.

[cit18] Moroz M. A., Kochetkov T., Cai S., Wu J., Shamis M., Nair J., De Stanchina E., Serganova I., Schwartz G. K., Banerjee D., Bertino J. R., Blasberg R. G. (2011). Clin. Cancer Res..

[cit19] Frisch D. J. , Trucks M. J., Schlegel G. W., Scuseria H. B., Robb G. E., Cheeseman M. A., Scalmani J. R., Barone G., Mennucci V., Petersson B., Nakatsuji G. A., Caricato H., Li M., Hratchian X., Izmaylov H. P., Bloino A. F., Zheng J. and Sonnenber G., Gaussian, Inc., Wallingford CT, 2009

[cit20] Pettersen E. F., Goddard T. D., Huang C. C., Couch G. S., Greenblatt D. M., Meng E. C., Ferrin T. E. (2004). J. Comput. Chem..

[cit21] Chung L. W., Sameera W. M. C., Ramozzi R., Page A. J., Hatanaka M., Petrova G. P., Harris T. V., Li X., Ke Z., Liu F., Li H. B., Ding L., Morokuma K. (2015). Chem. Rev..

[cit22] Kim D. W., Ahn D. S., Oh Y. H., Lee S., Kil H. S., Oh S. J., Lee S. J., Kim J. S., Ryu J. S., Moon D. H., Chi D. Y. (2006). J. Am. Chem. Soc..

[cit23] Leyton J., Smith G., Lees M., Perumal M., De Nguyen Q., Aigbirhio F. I., Golovko O., He Q., Workman P., Aboagye E. O. (2008). Mol. Cancer Ther..

[cit24] Leyton J., Alao J. P., Da Costa M., Stavropoulou A. V., Latigo J. R., Perumal M., Pillai R., He Q., Atadja P., Lam E. W. F., Workman P., Vigushin D. M., Aboagye E. O. (2006). Cancer Res..

[cit25] Heinzmann K., Nguyen Q. D., Honess D., Smith D. M., Stribbling S., Brickute D., Barnes C., Griffiths J., Aboagye E. (2018). J. Nucl. Med..

[cit26] he Huang S., Tang A., Drisco B., Zhang S. Q., Seeger R., Li C., Jong A. (1994). DNA Cell Biol..

[cit27] Ke P. Y., Kuo Y. Y., Hu C. M., Chang Z. F. (2005). Genes Dev..

[cit28] Hu C. M., Yeh M. T., Tsao N., Chen C. W., Gao Q. Z., Chang C. Y., Lee M. H., Fang J. M., Sheu S. Y., Lin C. J., Tseng M. C., Chen Y. J., Chang Z. F. (2012). Cancer Cell.

[cit29] Chan S. R., Salem K., Jeffery J., Powers G. L., Yan Y., Shoghi K. I., Mahajan A. M., Fowler A. M. (2018). J. Nucl. Med..

[cit30] Coenen H. H., Gee A. D., Adam M., Antoni G., Cutler C. S., Fujibayashi Y., Jeong J. M., Mach R. H., Mindt T. L., Pike V. W., Windhorst A. D. (2017). Nucl. Med. Biol..

[cit31] Yun M., Oh S. J., Ha H. J., Ryu J. S., Moon D. H. (2003). Nucl. Med.
Biol..

[cit32] Jia Z. J., Kelberlau S., Olsson L., Anilkumar G., Fraser-Reid B. (1999). Synlett.

[cit33] Hsu P. D., Lander E. S., Zhang F. (2014). Cell.

[cit34] Stella S., Alcon P., Montoya G. (2017). Nat. Struct. Mol. Biol..

[cit35] Allott L., Miranda C., Hayes A., Raynaud F., Cawthorne C., Smith G. (2019). EJNMMI Radiopharm. Chem..

[cit36] Workman P., Aboagye E. O., Balkwill F., Balmain A., Bruder G., Chaplin D. J., Double J. A., Everitt J., Farningham D. A. H., Glennie M. J., Kelland L. R., Robinson V., Stratford I. J., Tozer G. M., Watson S., Wedge S. R., Eccles S. A., Navaratnam V., Ryder S. (2010). Br. J. Cancer.

