# Supplementary material for: Synthesis and evaluation of 3′-[18F]fluorothymidine-5′-squaryl as a bioisostere of 3′-[18F]fluorothymidine-5′-monophosphate
Source: RSC Adv. 2021 Mar 29;11(20):12423–33. doi: 10.1039/d1ra00205h (PMC8696986; doi:10.1039/d1ra00205h)
Supplement: RA-011-D1RA00205H-s001 [file RA-011-D1RA00205H-s001.pdf]

Electronic Supplementary Material (ESI) for RSC Advances.
This journal is © The Royal Society of Chemistry 2021

Synthesis and evaluation of 3'-[^{18}F]fluorothymidine-5'-squaryl as a bioisostere of 3'-[^{18}F]fluorothymidine-5'-monophosphate

D. Brickute[†a], A. Beckley[†a], L. Allott[a], M. Braga[a], C. Barnes[a], K. J. Thorley[b] and E. O. Aboagye[a*]

[a] Comprehensive Cancer Imaging Centre, Faculty of Medicine, Imperial College London, Hammersmith Hospital, W12 0NN, London, UK; [b] University of Kentucky, Department of Chemistry, Lexington KY 40506, USA

Supporting Information

Contents

1.0 Chemistry……..Page 01
2.0 NMR Spectra……..Page 02
3.0 Radiochemistry……..Page 15
4.0 HPLC Chromatograms……..Page16
5.0 LogD$_{7.5}$ determination……..Page 18
6.0 Metabolite analysis in human liver microsomes……..Page19
7.0 Gene structure and positions of CRISPR and PCR primers……..Page 19
8.0 Determining if [^{18}F]SqFLT is a substrate for TK1 and ENT1/2……..Page 19
9.0 Determining if [^{18}F]SqFLT is a substrate for MDR proteins……..Page 20
10.0 Time activity curves (TAC)……..Page 20
11.0 References……..Page 20

1.0 Chemistry

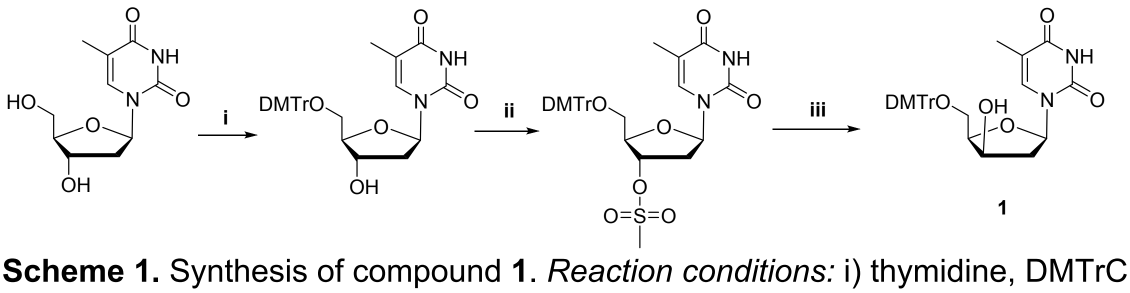

1

Scheme 1. Synthesis of compound **1**. *Reaction conditions:* i) thymidine, DMTrCl, pyridine, RT, 16 h; ii) MsCl, 0 °C, 3 h; iii) NaOH (10 N), EtOH, 80 °C, 1.5 h.

Compound **1** was synthesised according to literature procedures.[1,2]

2.0 NMR Spectra

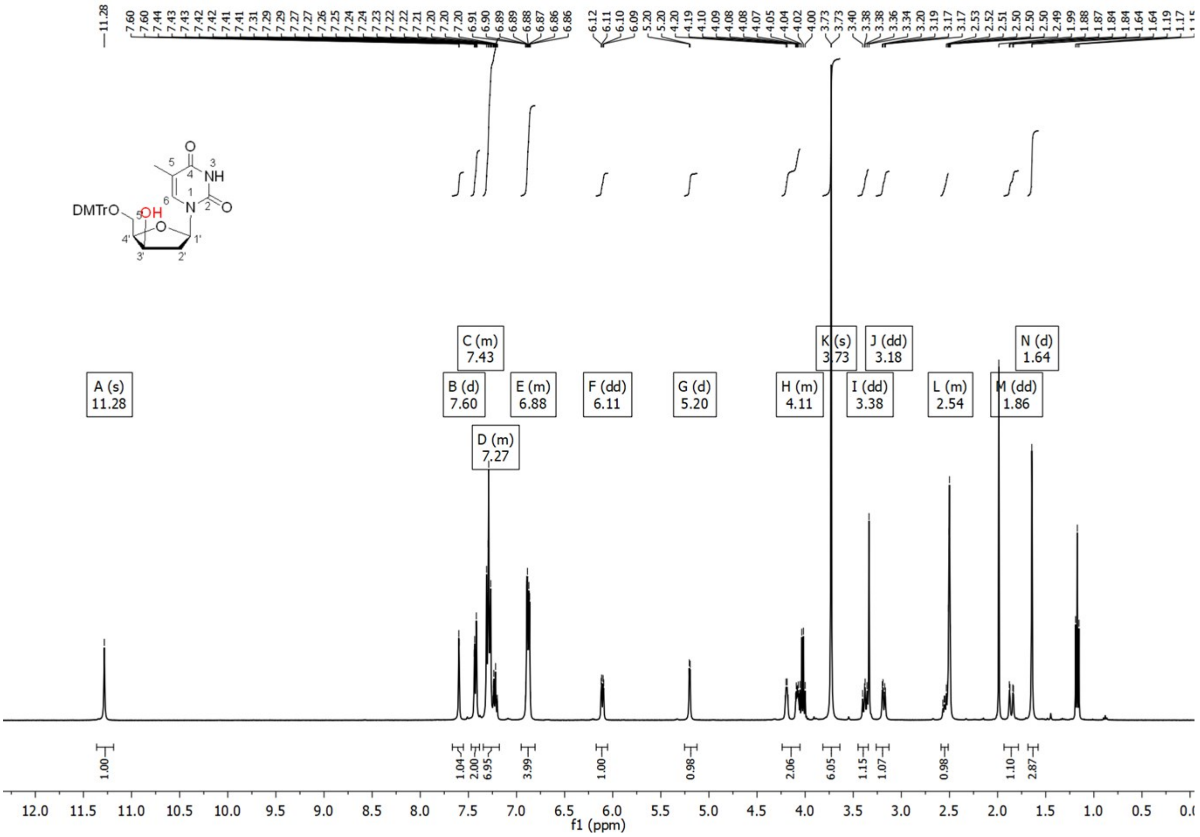

Figure 1. ^{1}H-NMR spectra of compound **1**.

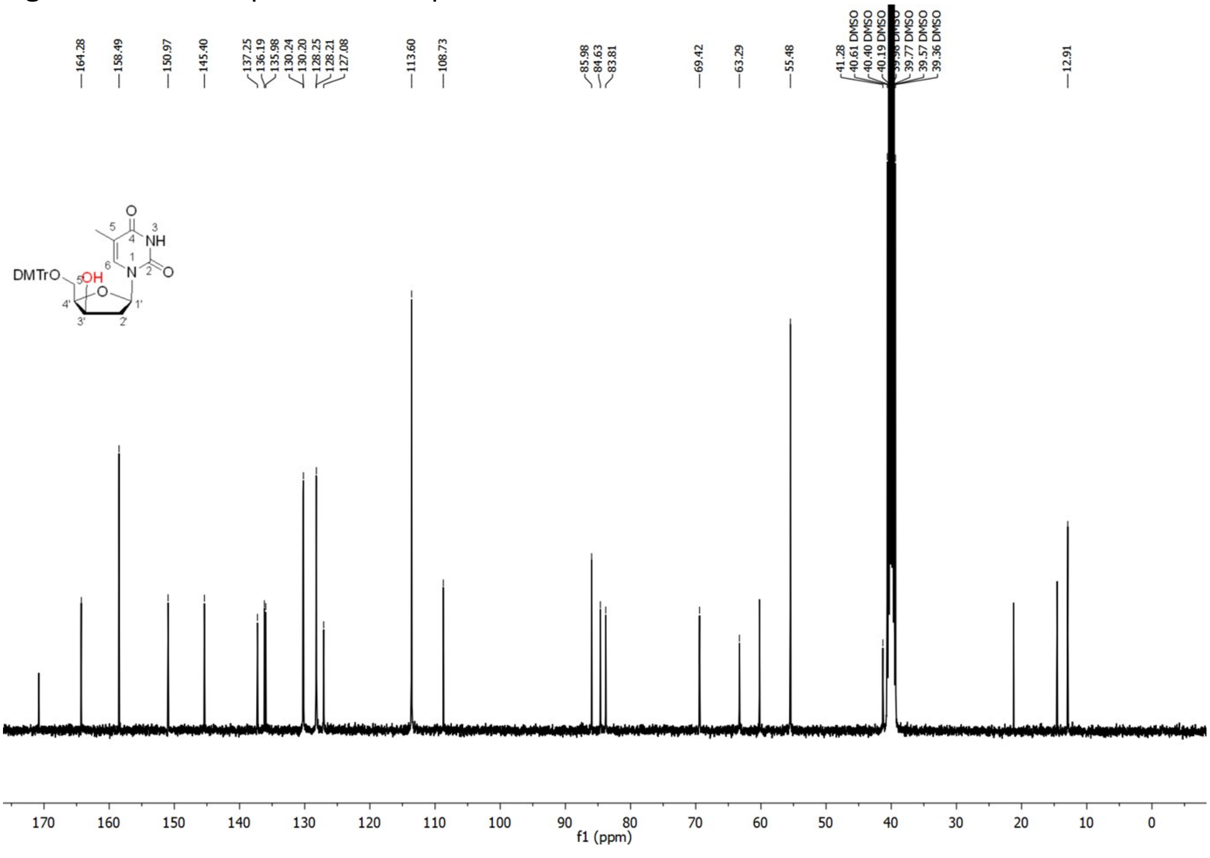

Figure 2. ^{13}C-NMR spectra of compound **1.**

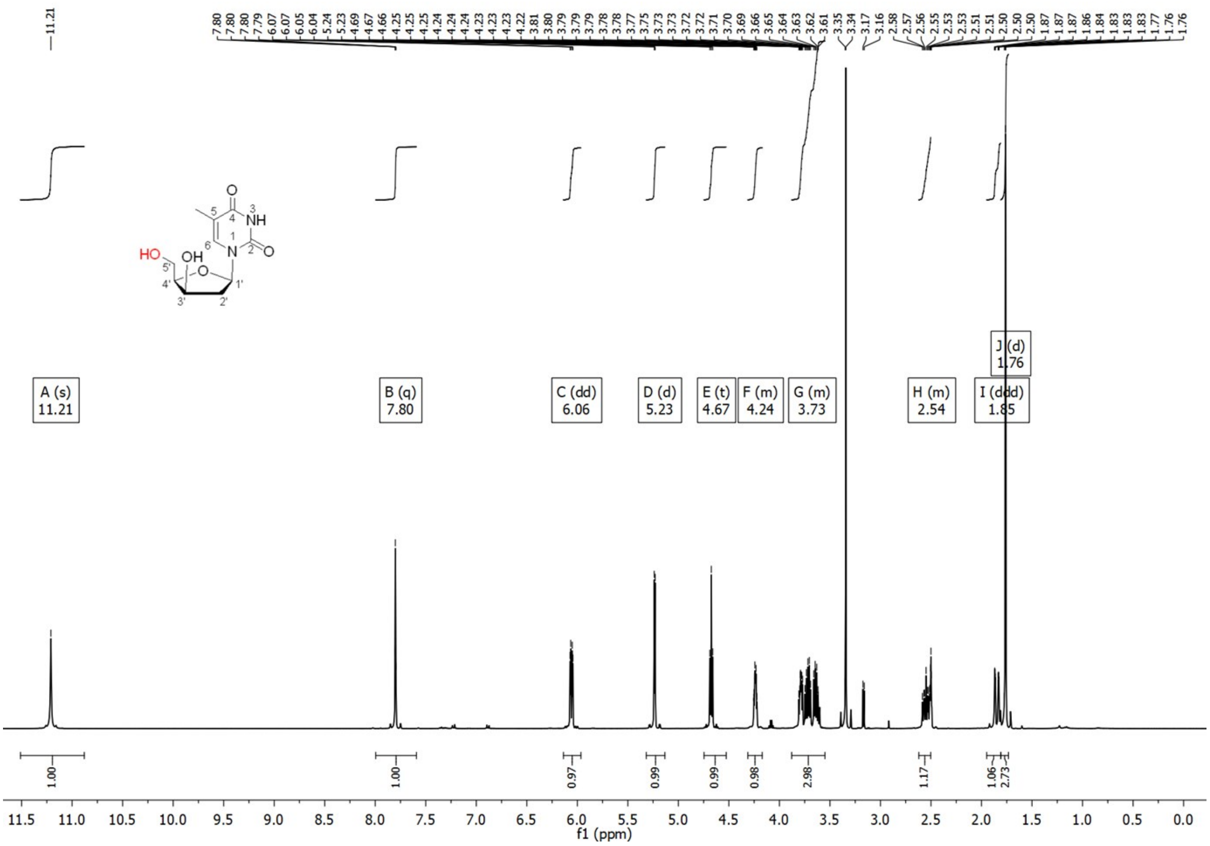

Figure 3. ^{1}H-NMR spectra of compound **2**.

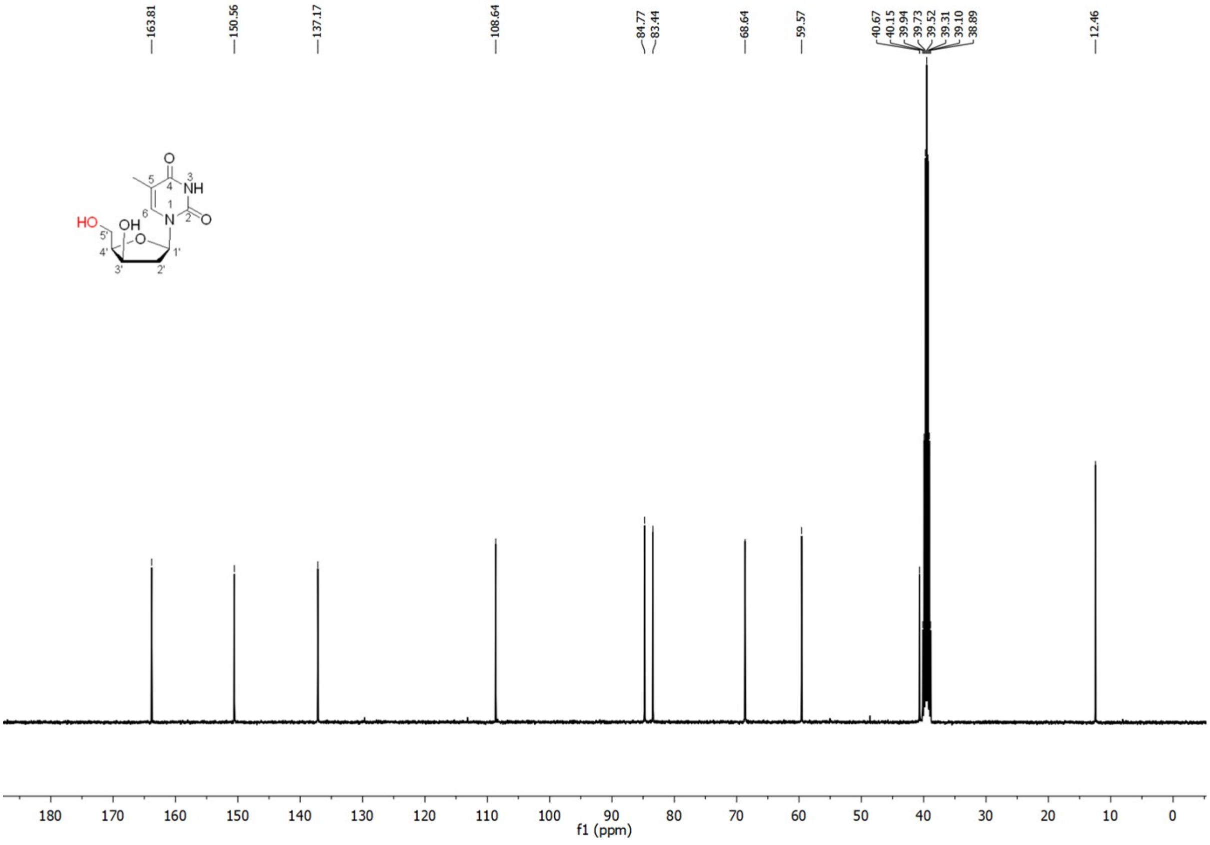

Figure 4. ^{13}C-NMR spectra of compound **2**.

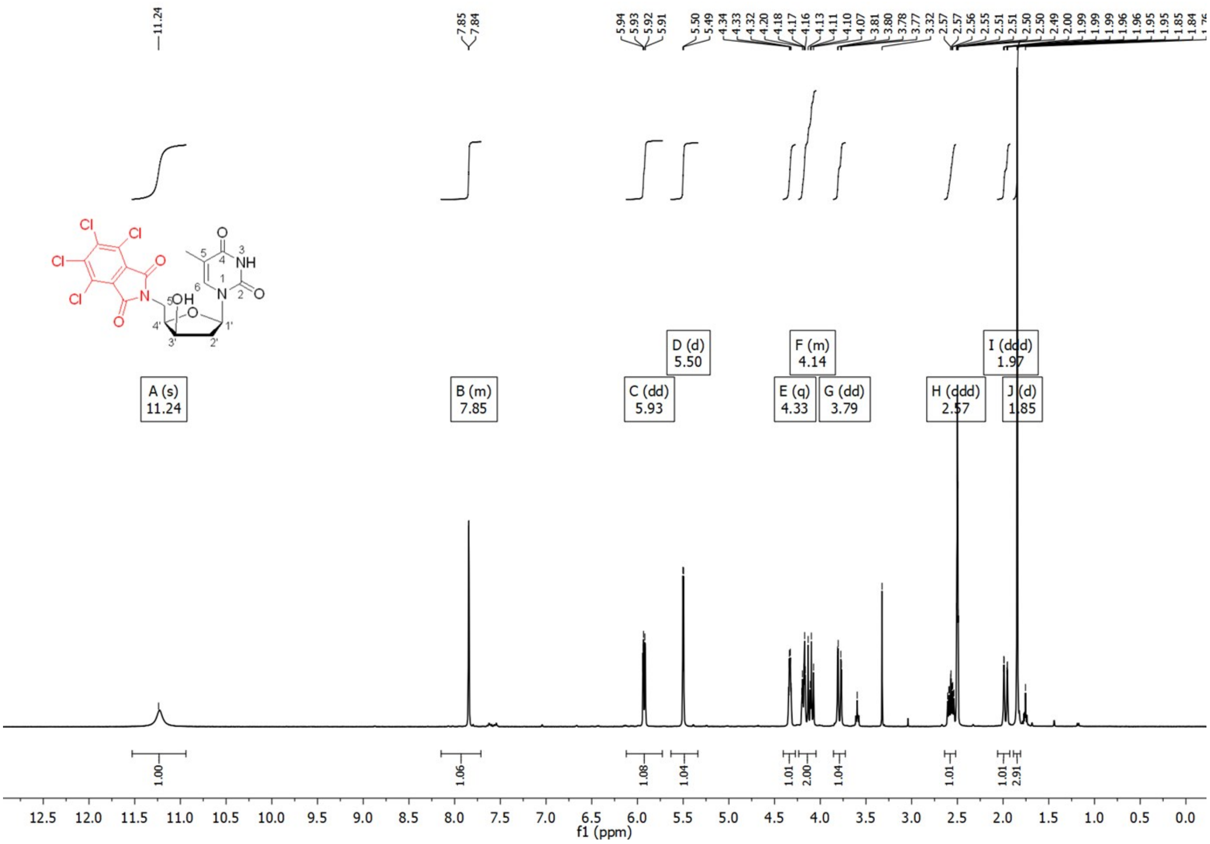

Figure 5. ^{1}H-NMR spectra of compound **3**.

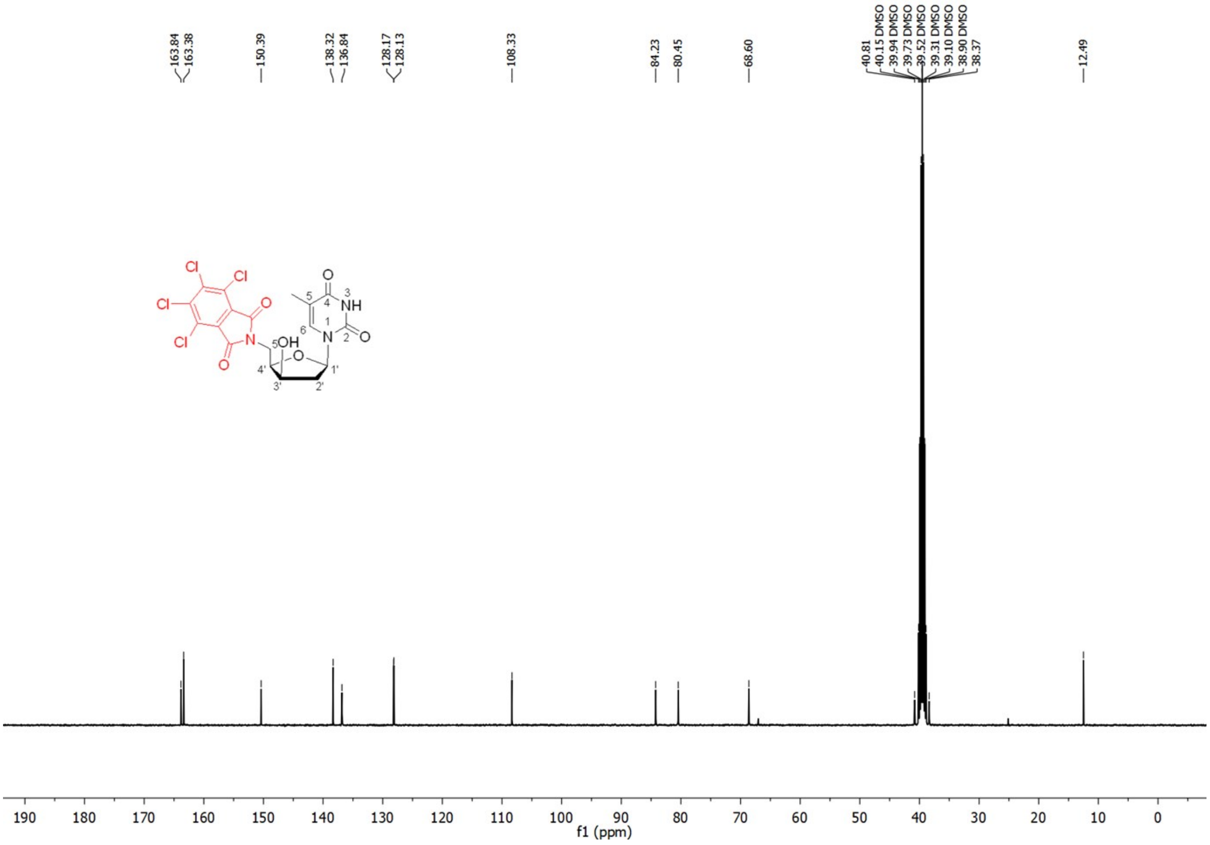

Figure 6. ^{13}C-NMR spectra of compound **3**.

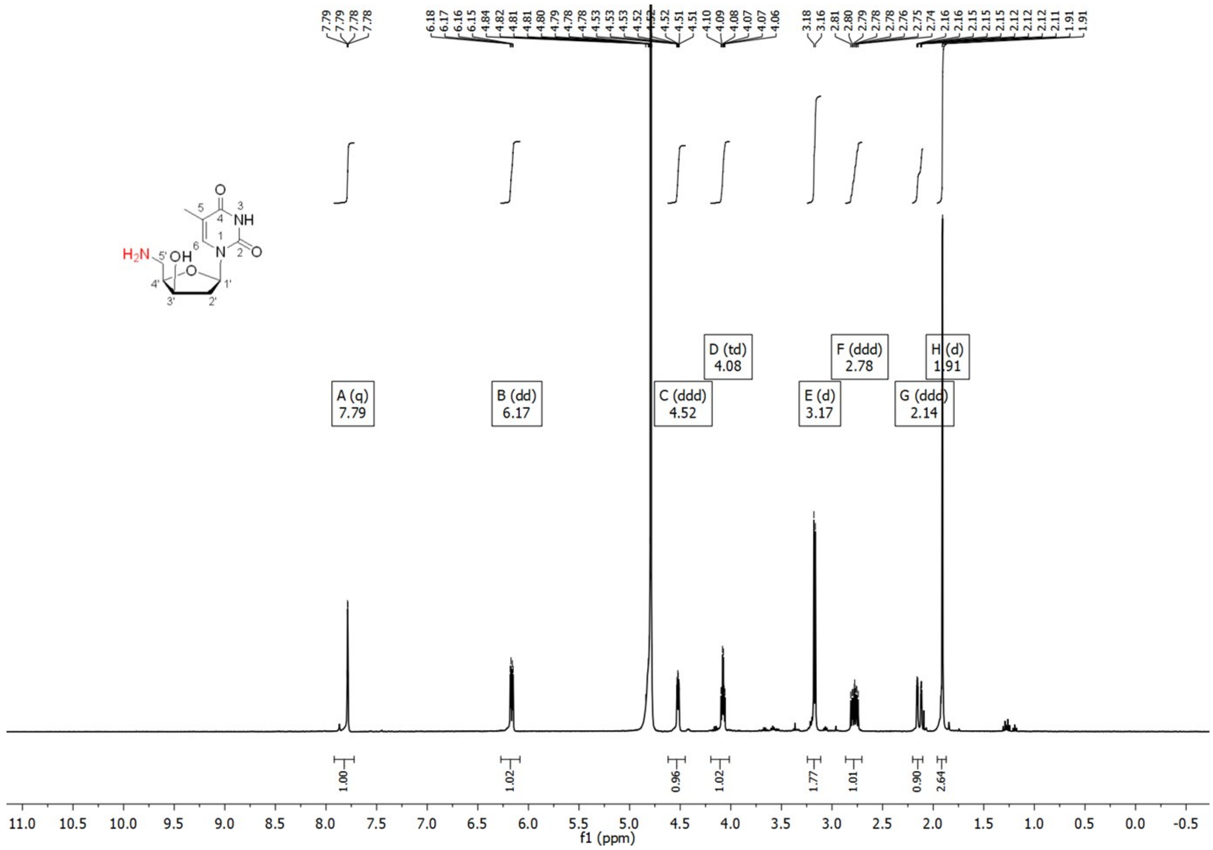

Figure 7. ^{1}H-NMR spectra of compound **4**.

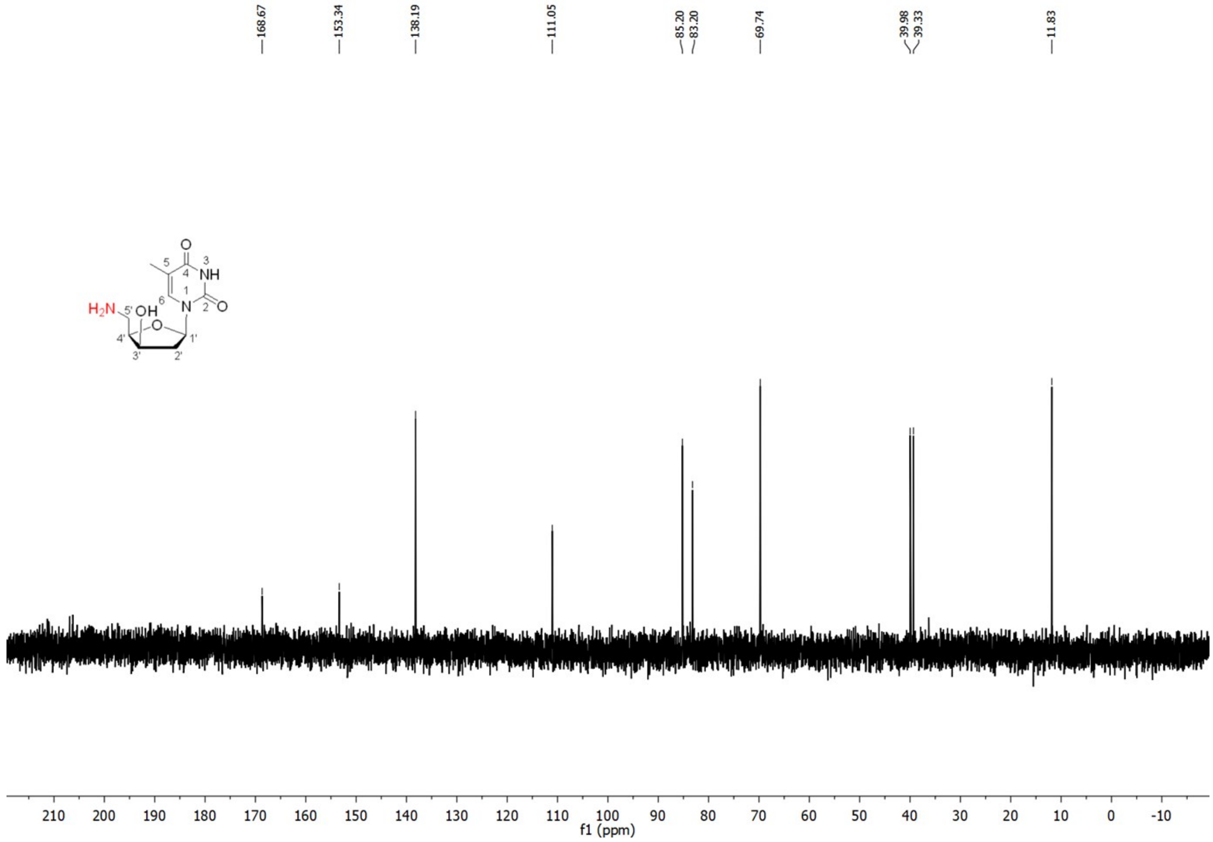

Figure 8. ^{13}C-NMR spectra of compound **4**.

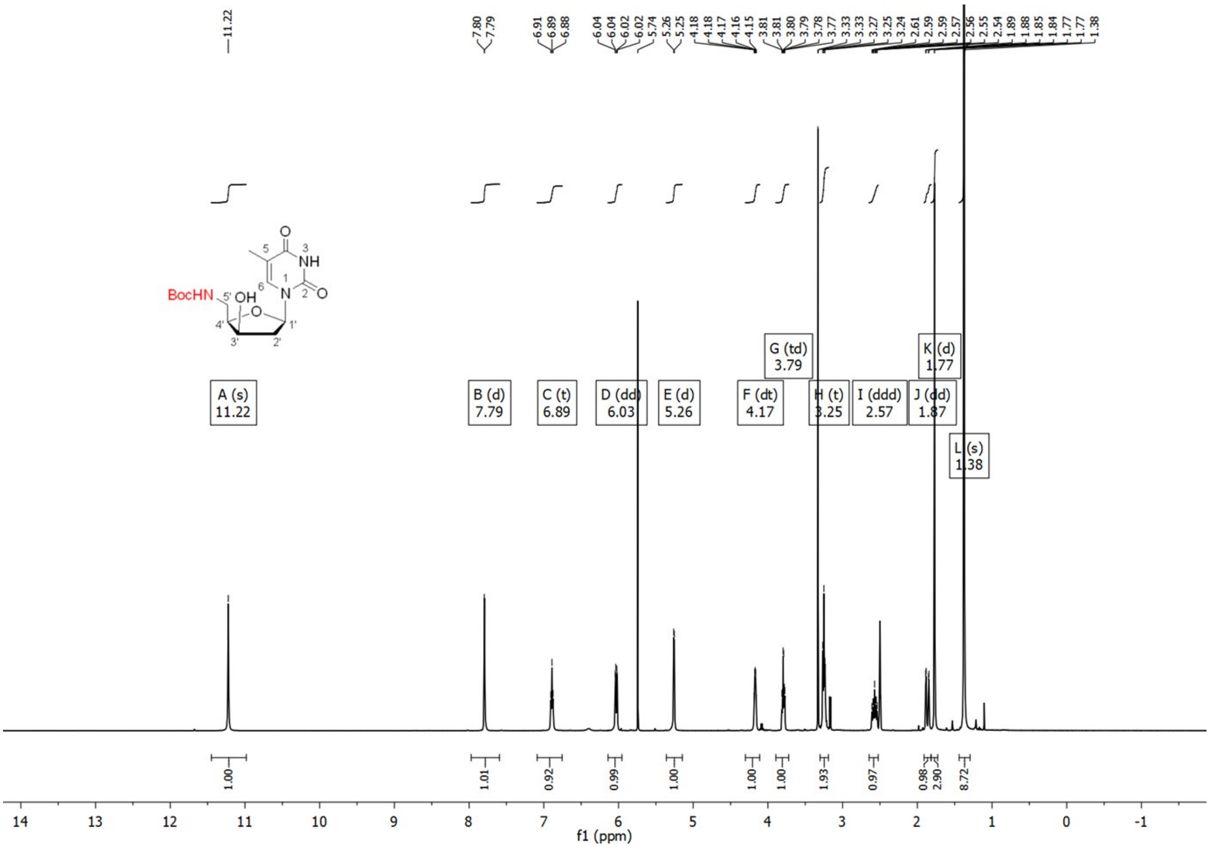

Figure 9. ^{1}H-NMR spectra of compound **5**.

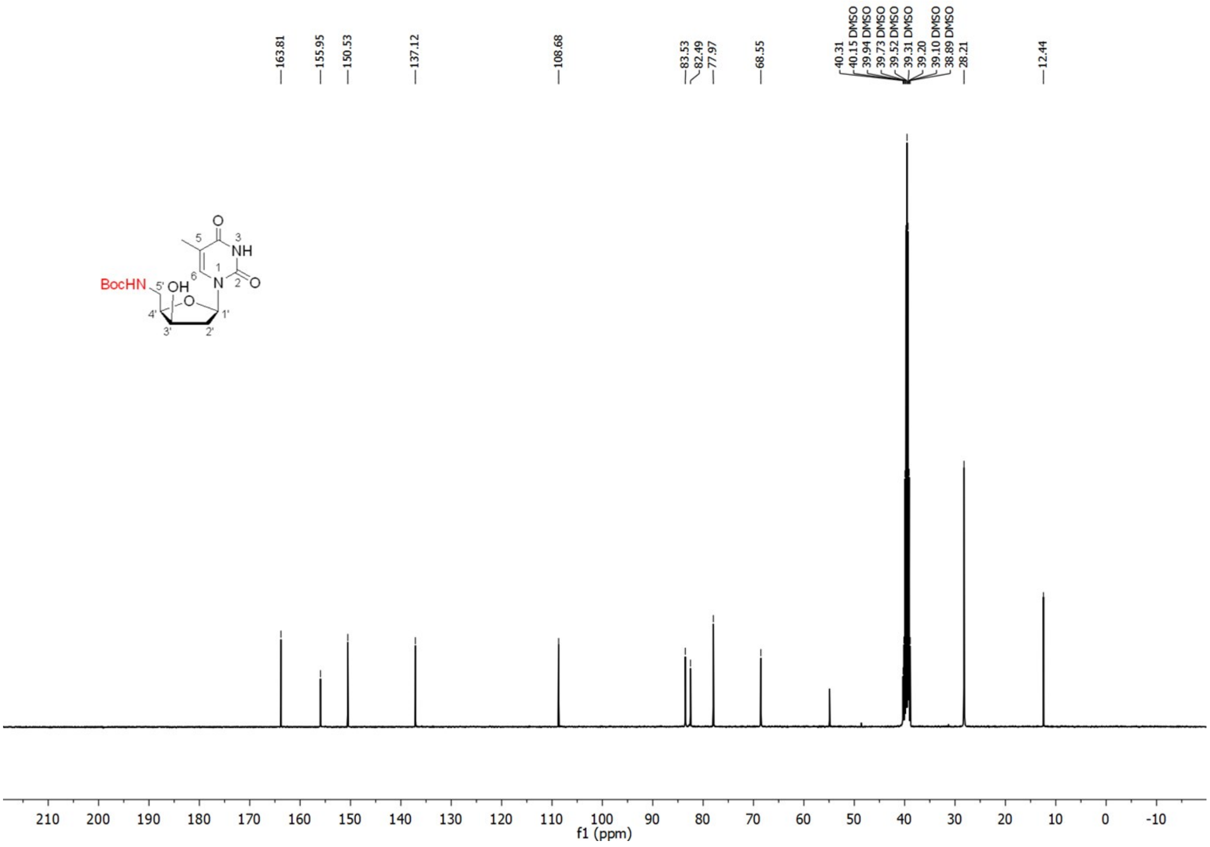

Figure 10. ^{13}C-NMR spectra of compound **5**.

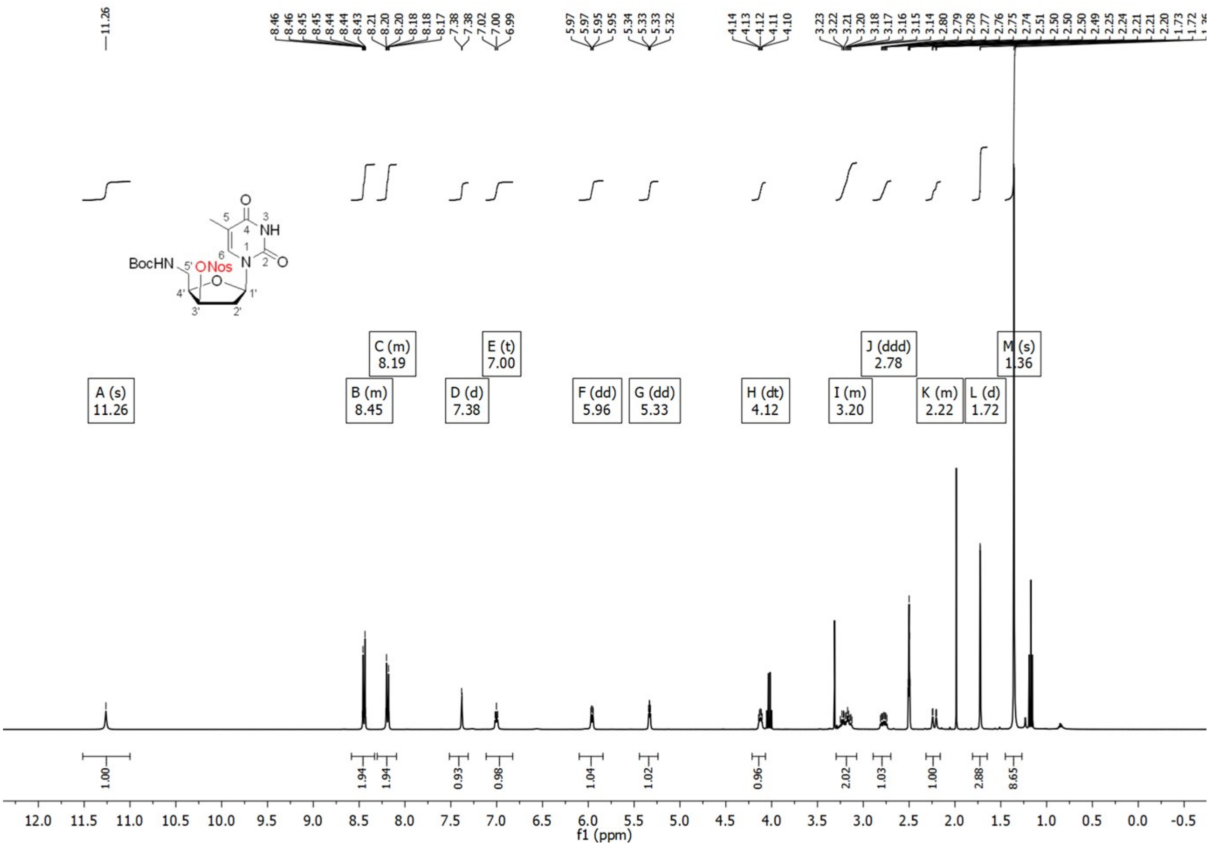

Figure 11. ^{1}H-NMR spectra of compound **6**.

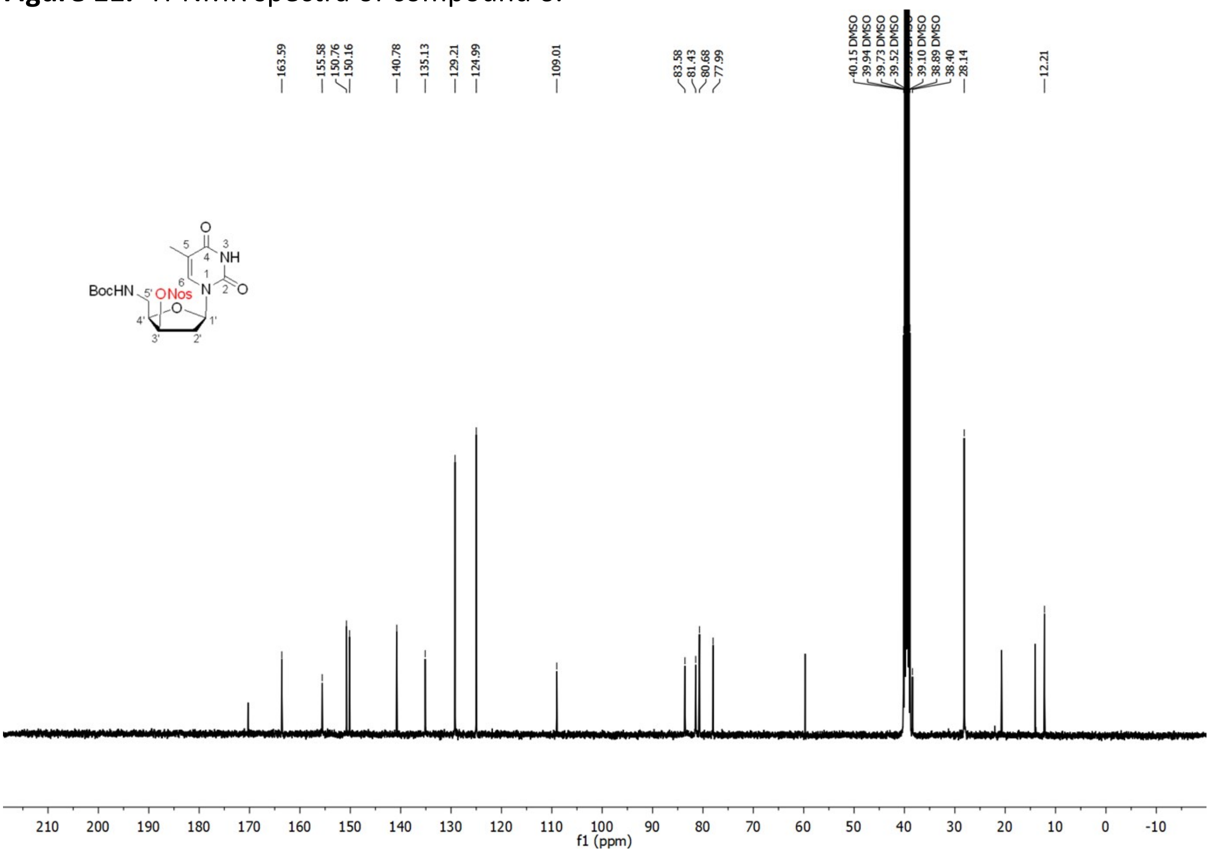

Figure 12. ^{13}C-NMR spectra of compound **6**.

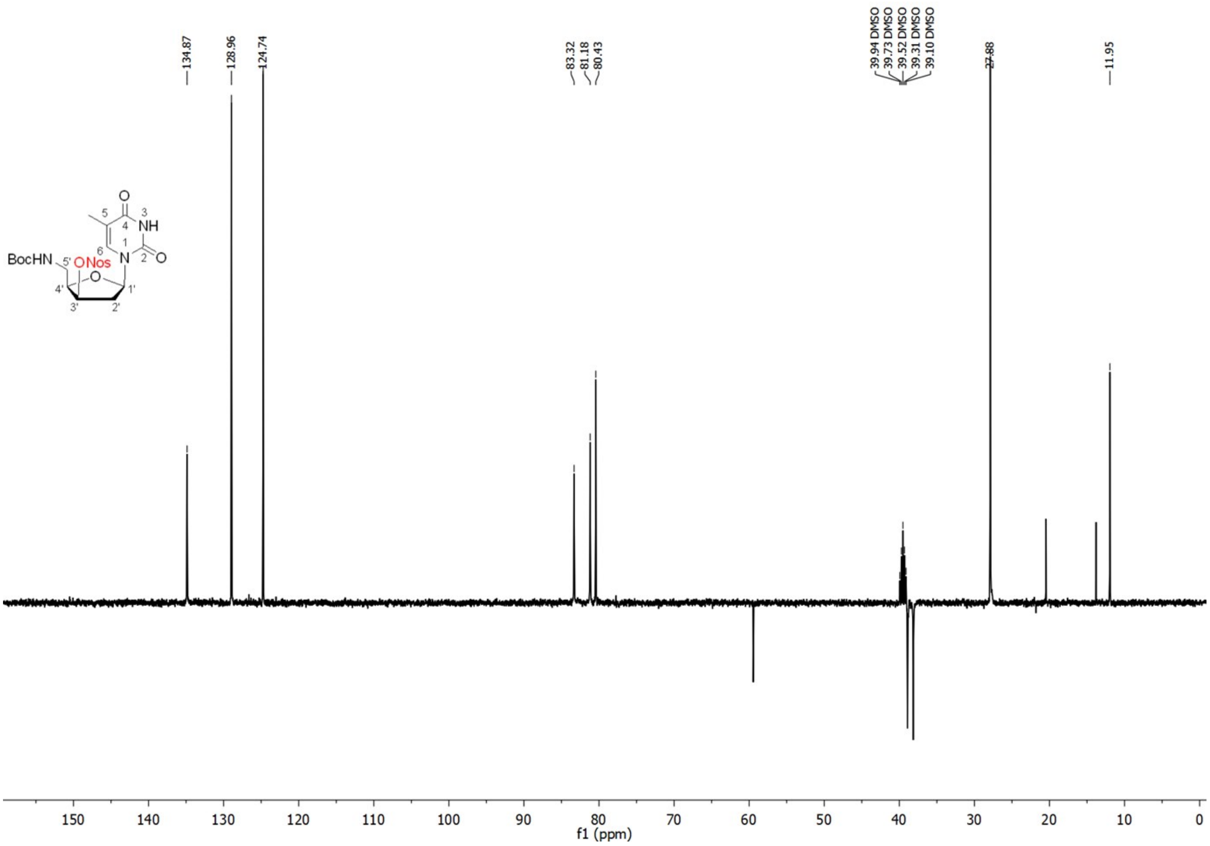

Figure 13. ^{13}C-DEPT-NMR spectra of compound **6**.

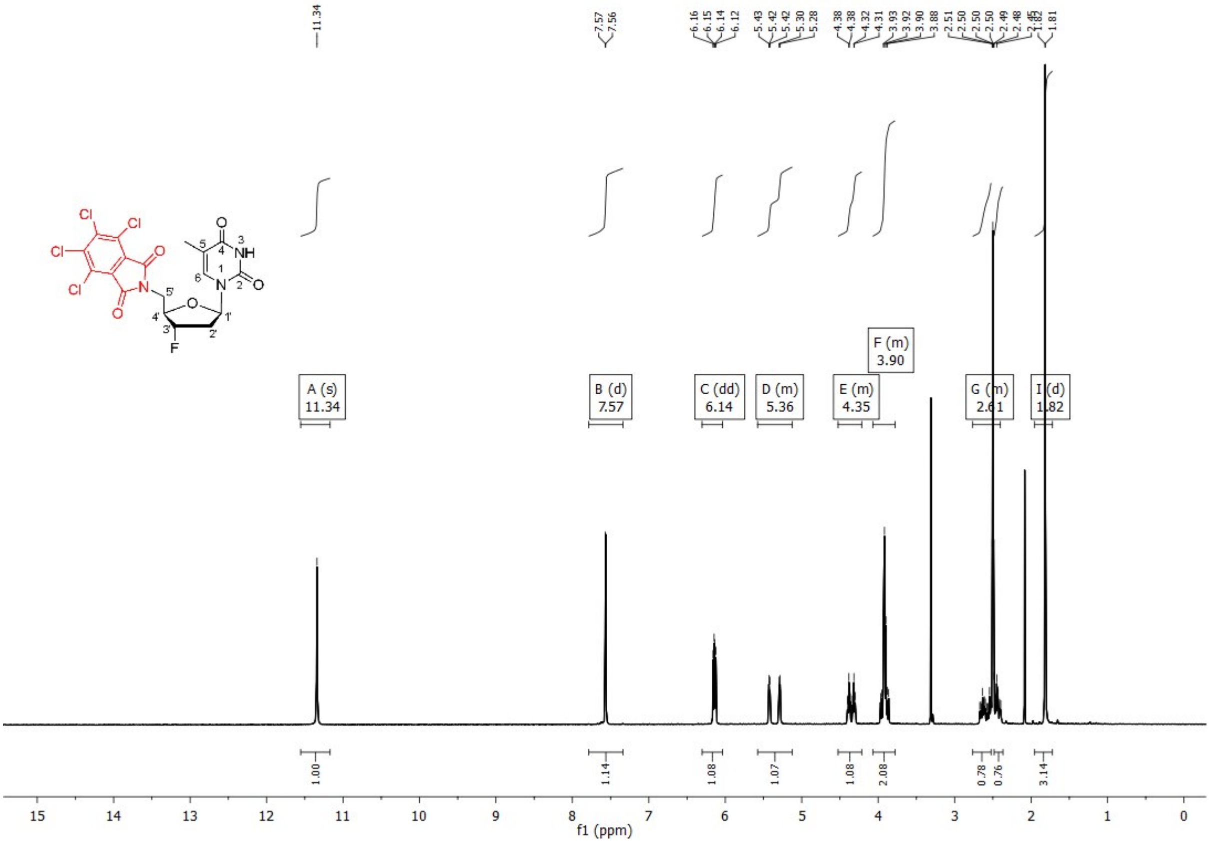

Figure 14. ^{1}H-NMR spectra of compound **8**.

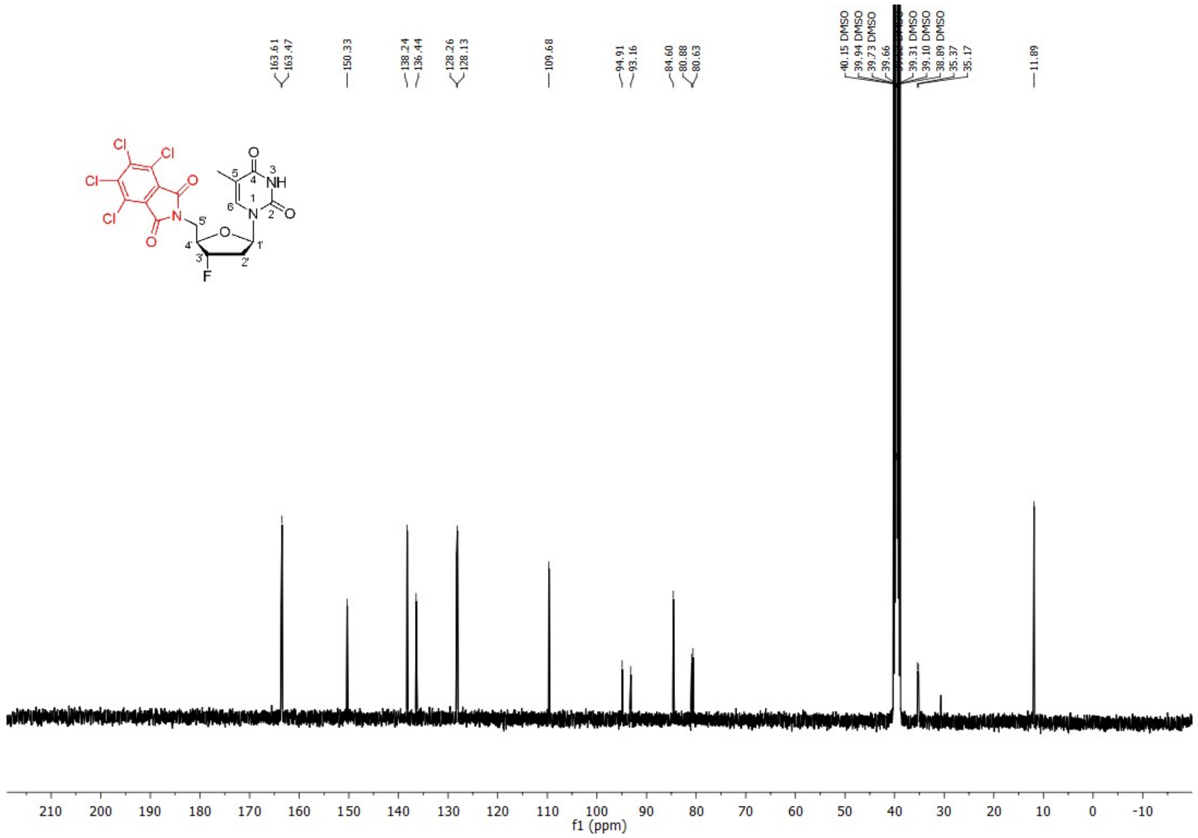

Figure 15. ^{13}C-NMR spectra of compound **8**.

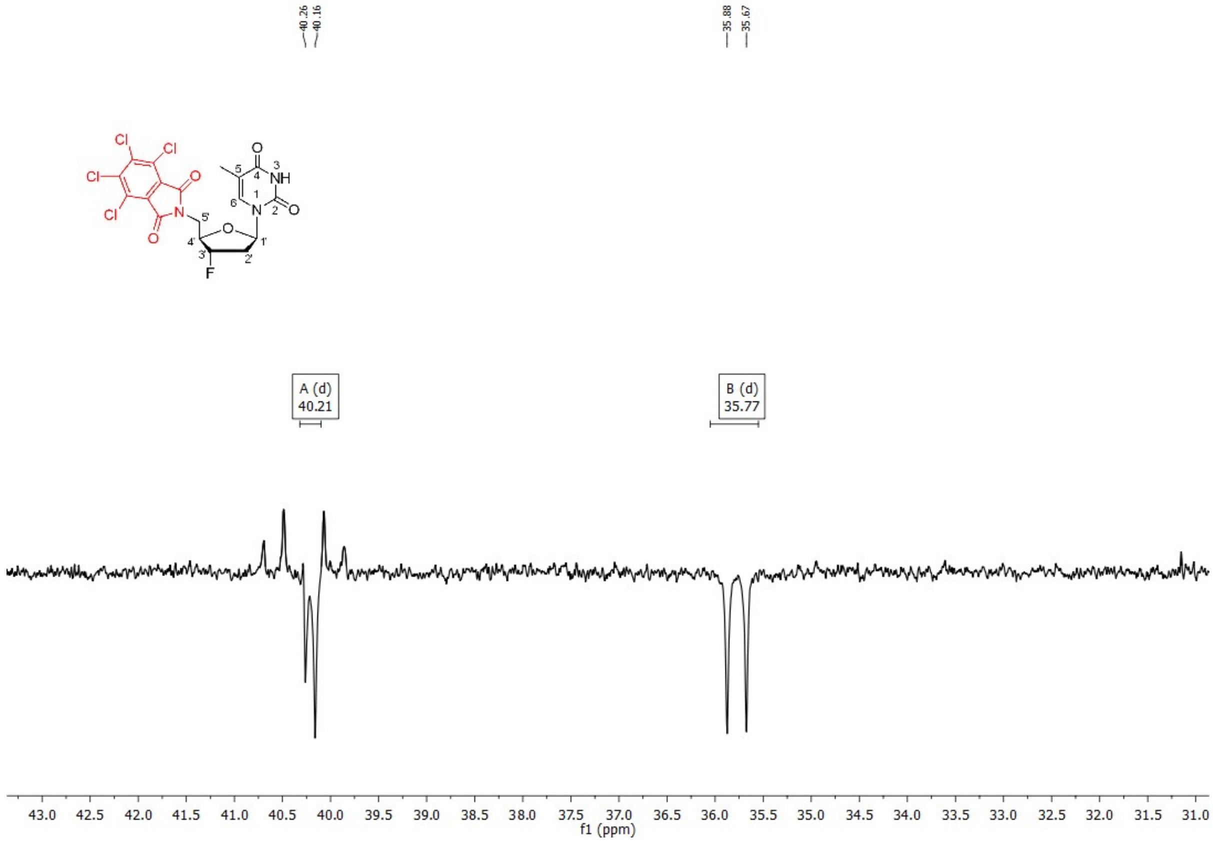

Figure 16. DEPT-NMR spectra of compound **8**, showing carbon peaks that would otherwise be hidden by the DMSO solvent peak.

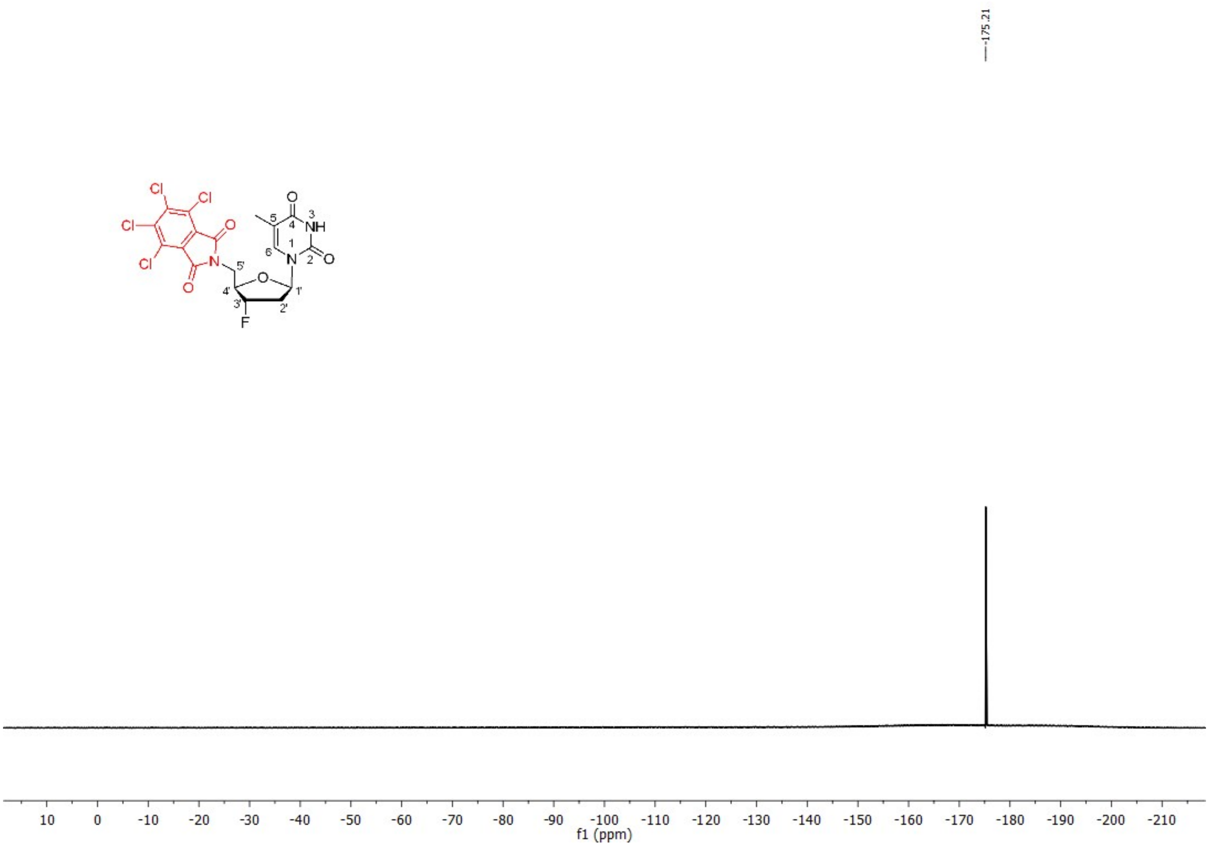

Figure 17. ^{19}F-NMR spectra of compound **8**.

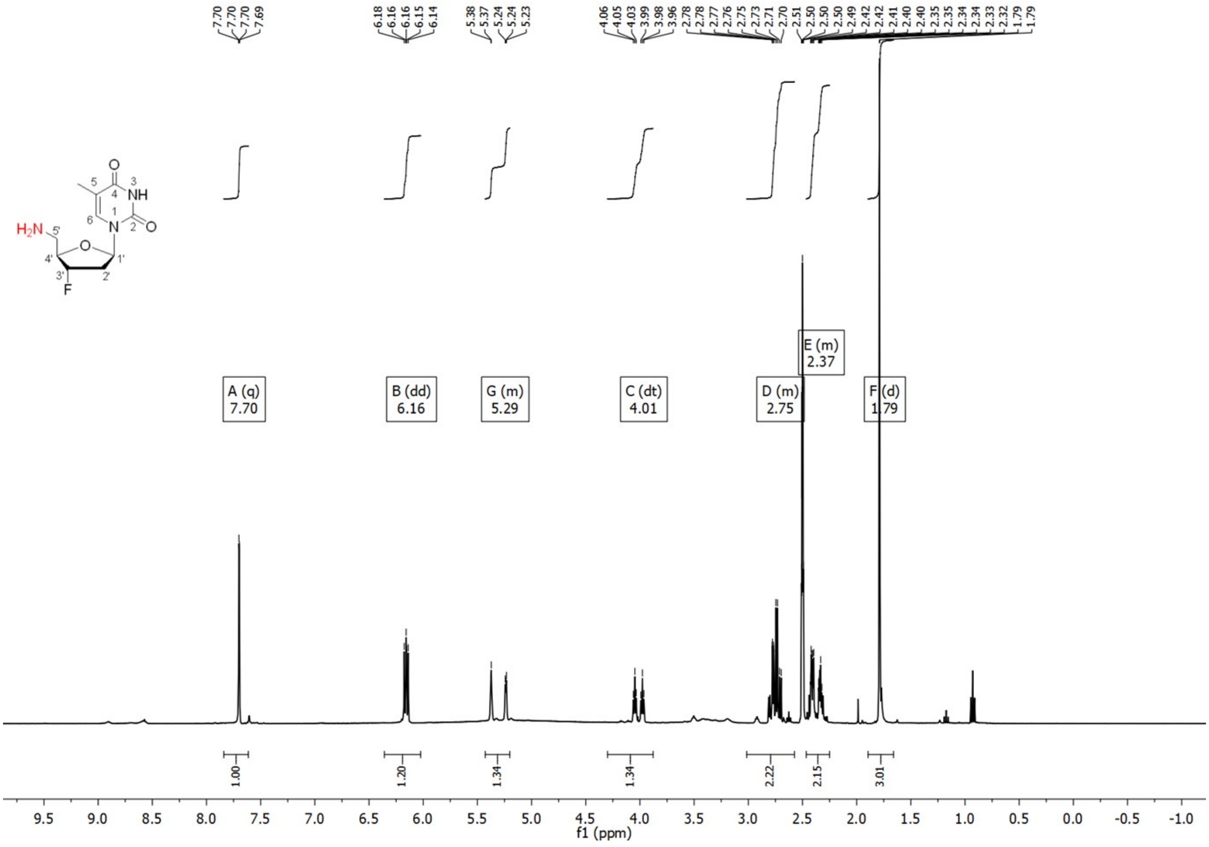

Figure 18. ^{1}H-NMR spectra of compound **9**.

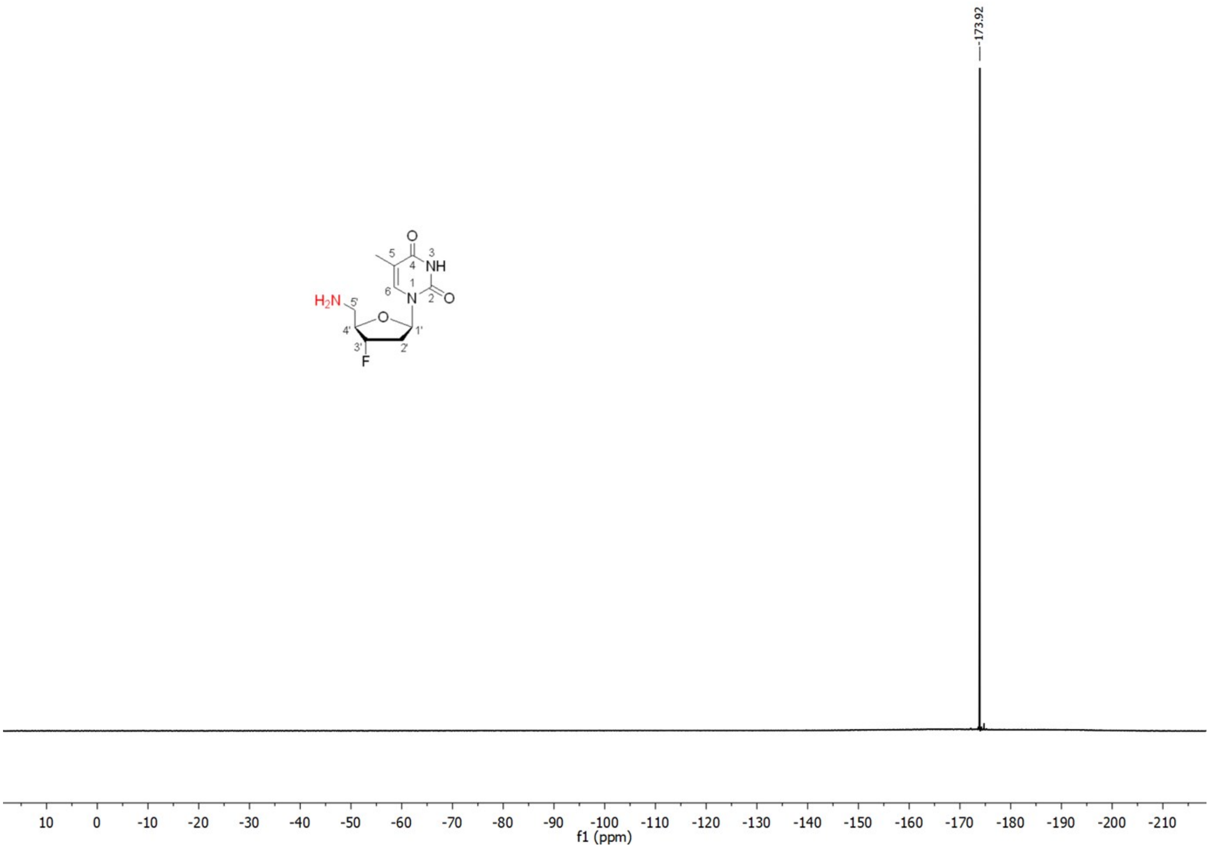

Figure 19. ^{19}F-NMR spectra of compound **9**.

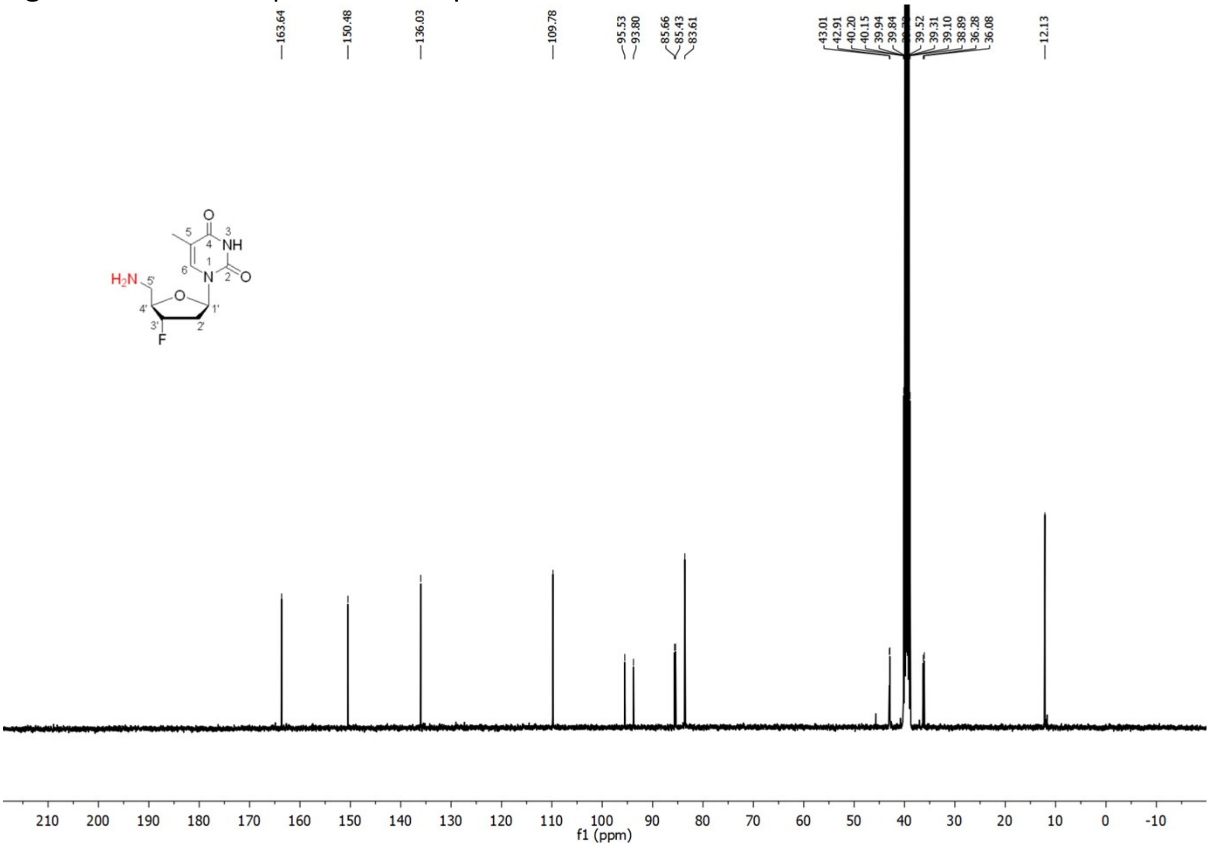

Figure 20. ^{13}C-NMR spectra of compound **9**.

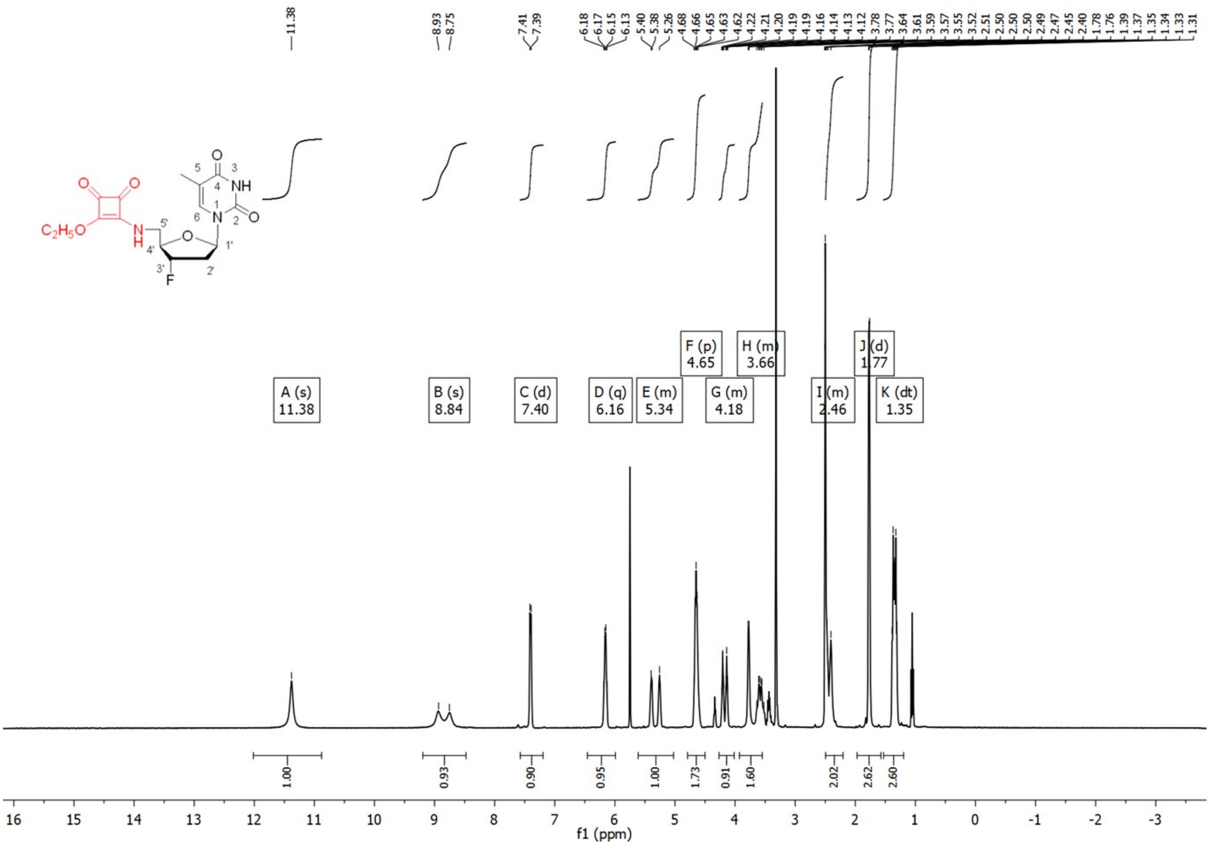

Figure 21. ^{1}H-NMR spectra of compound **10**.

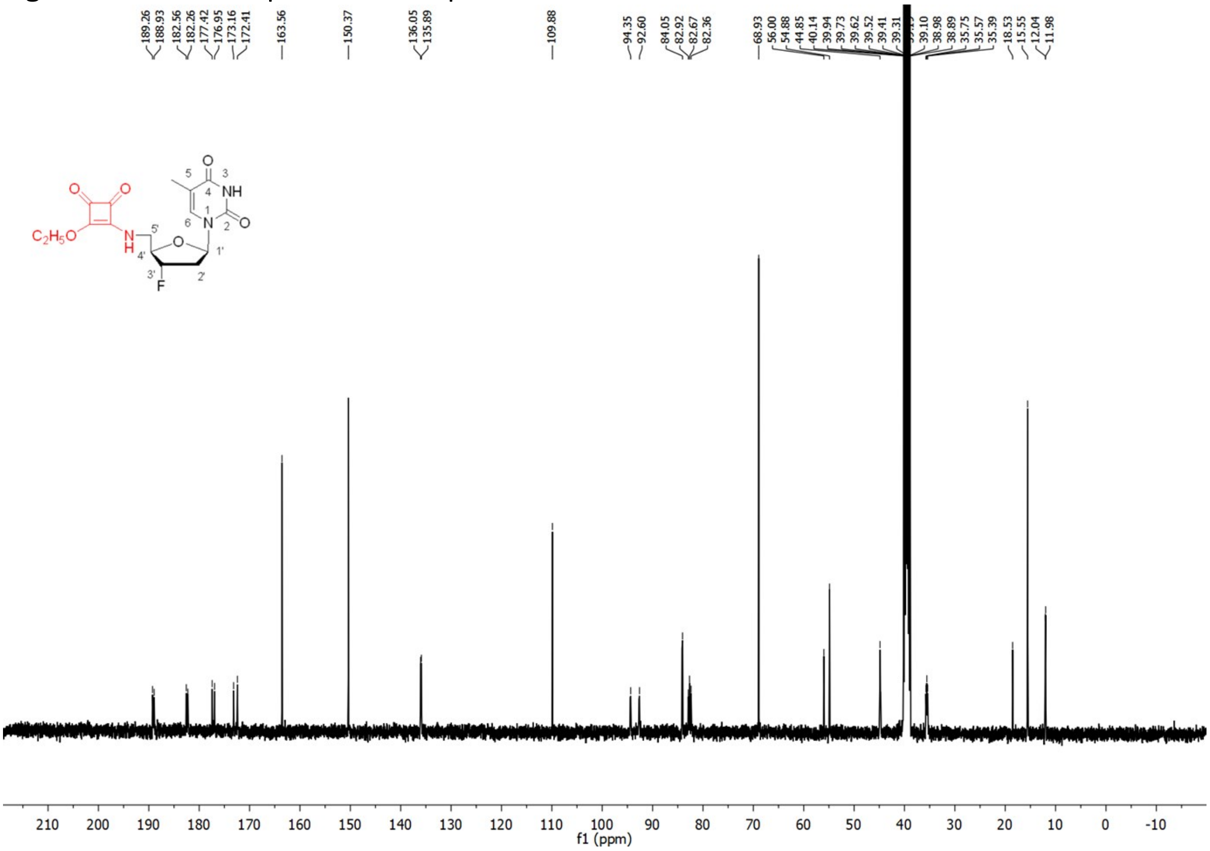

Figure 22. ^{13}C-NMR spectra of compound **10**.

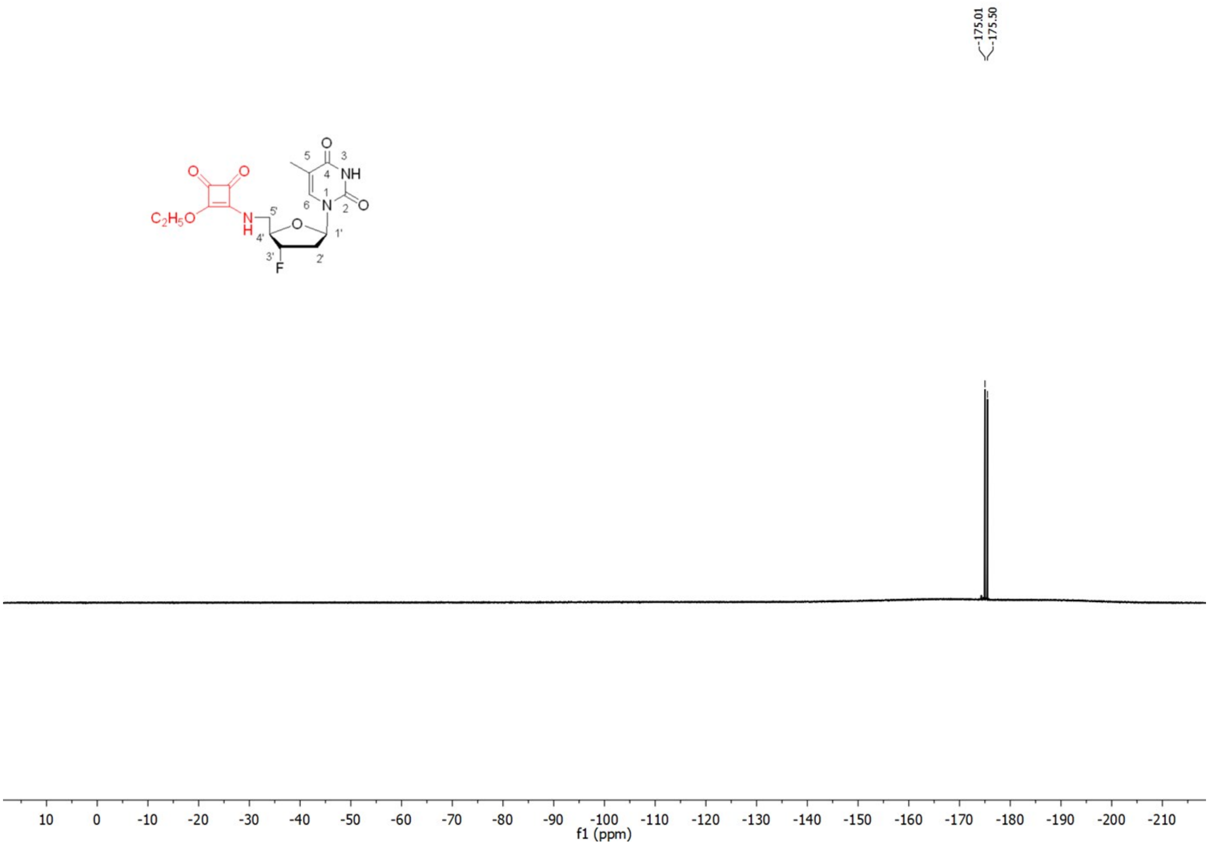

Figure 23. ^{19}F-NMR spectra of compound **10**.

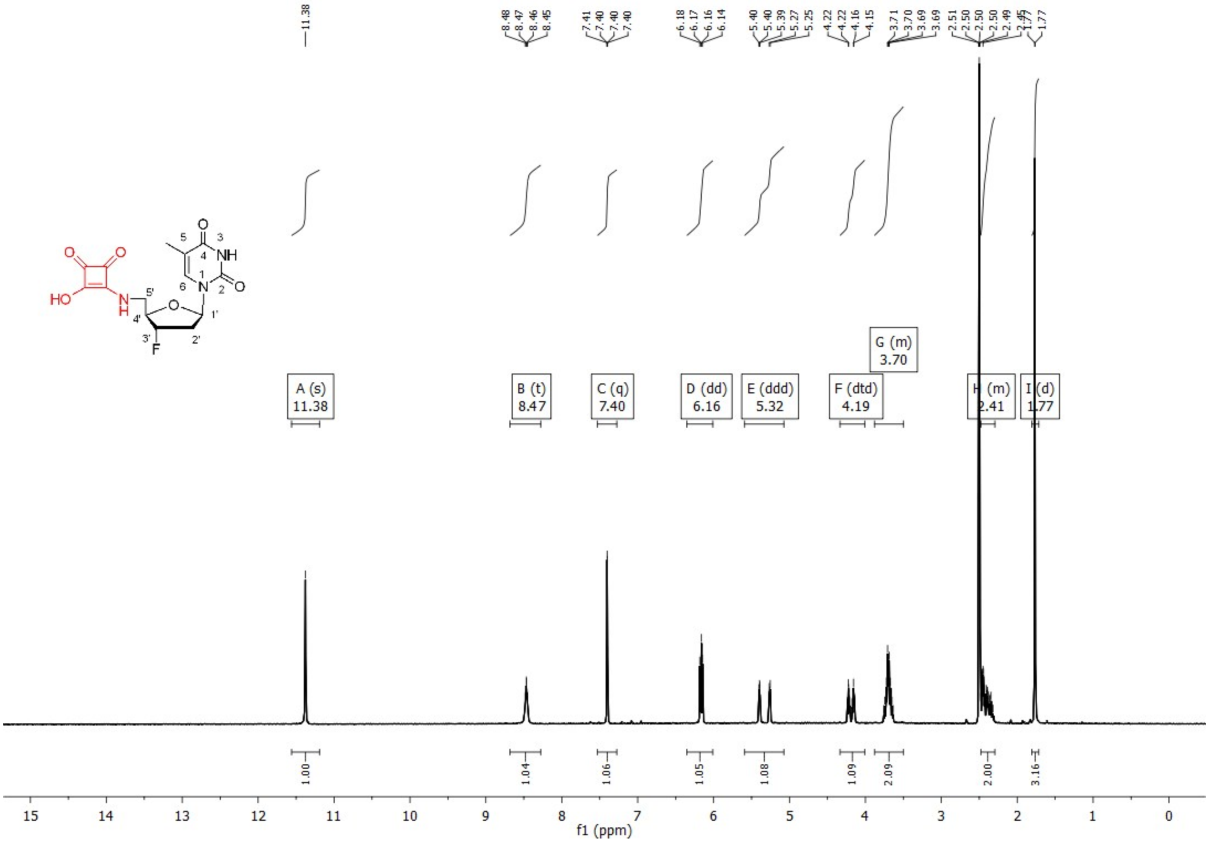

Figure 24. ^{1}H-NMR spectra of compound **11**.

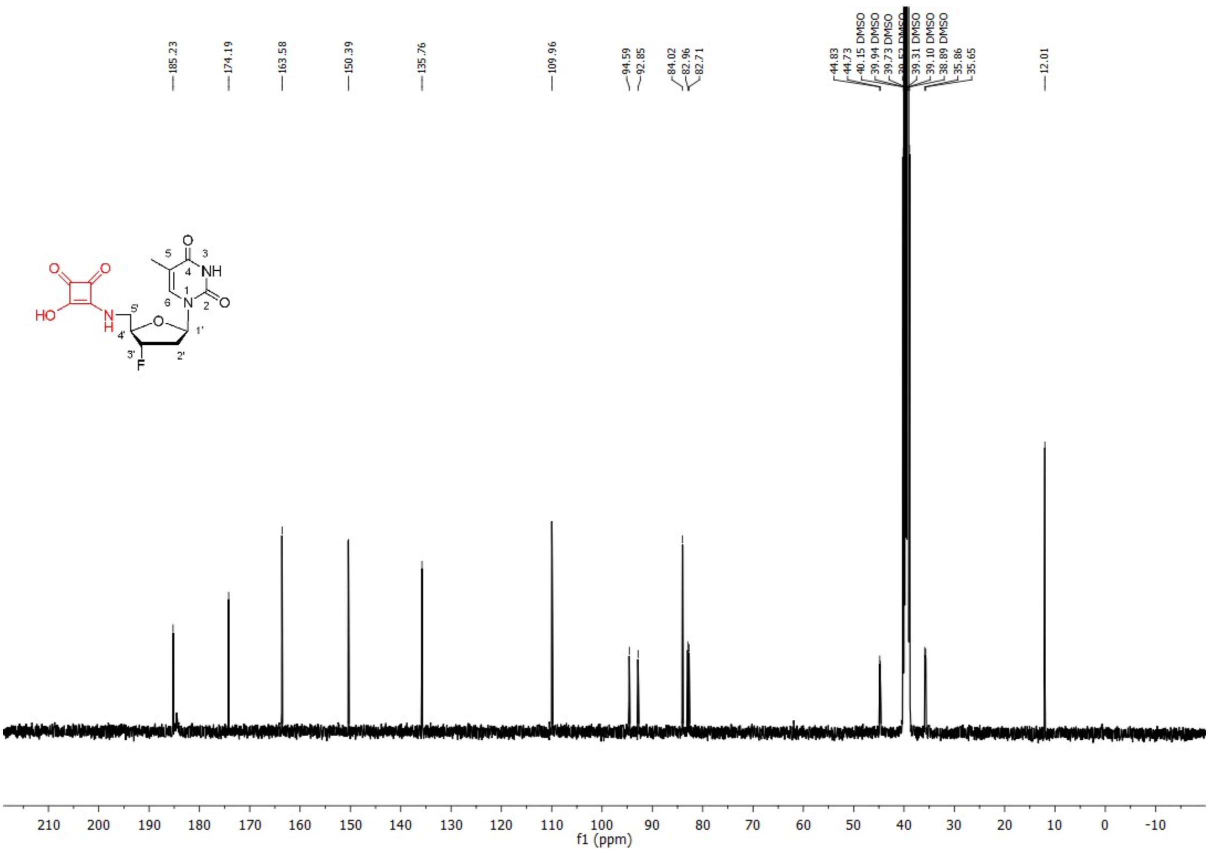

Figure 25. ^{13}C-NMR spectra of compound **11**.

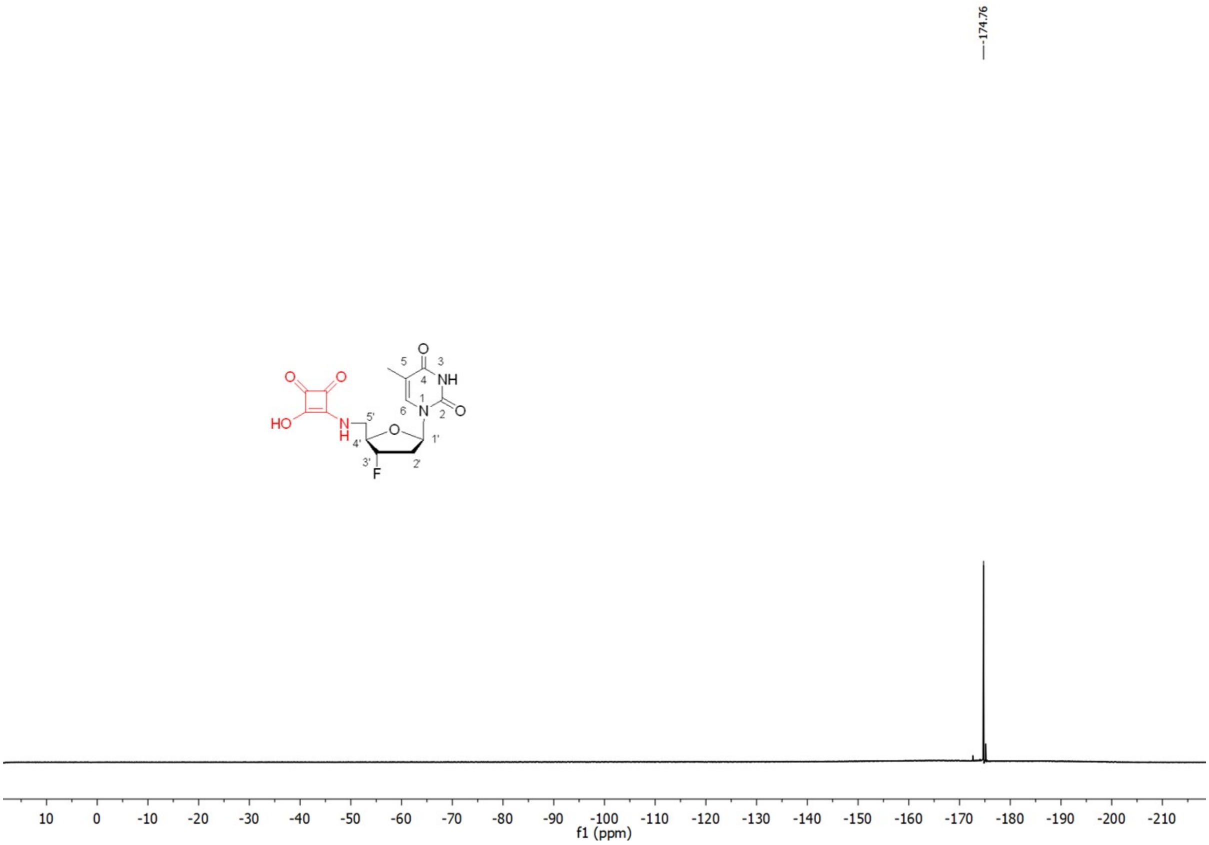

Figure 26. ^{19}F-NMR spectra of compound **11**.

3.0 Radiochemistry

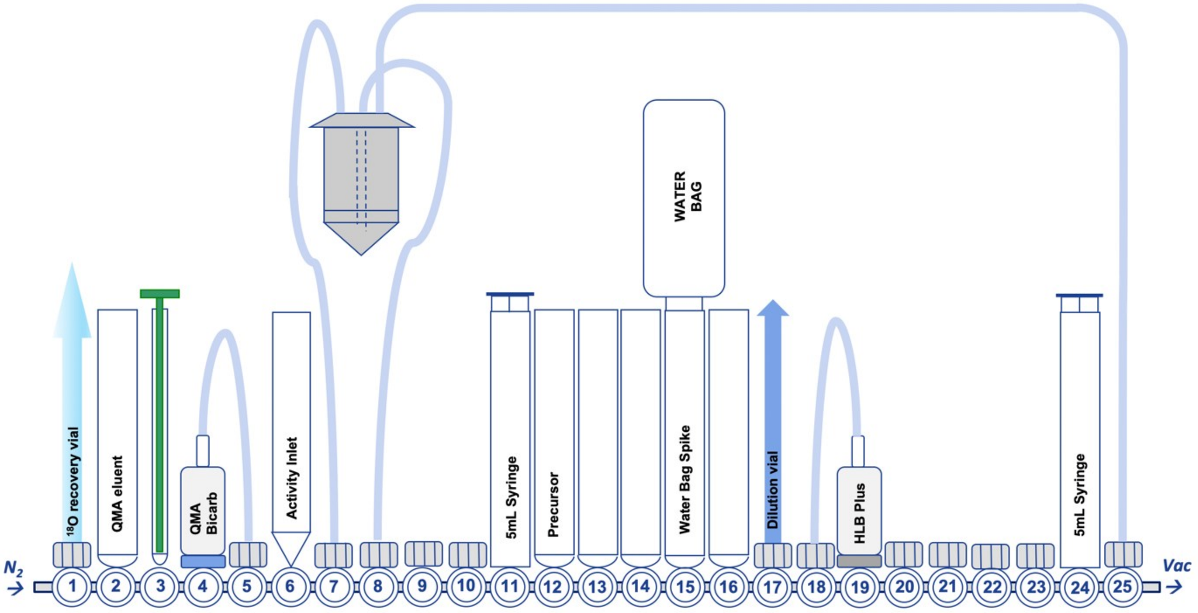

Position	Description
2	**QMA Eluent:** TBAHC (160 uL) and MeCN (640 uL)
4-5	QMA Bicarbonate SPE Cartridge
12	**Precursor:** Compound **6** (12 mg) dissolved in 2-methyl-2-butanol/MeCN (1:1 v/v, 1.2 mL)
15	Water Bag
17	**Dilution Vial:** Water (40 mL)
18 - 19	HLB Plus SPE Cartridge – to trap compound **[^{18}F]7**

Figure 27. A schematic representation of the GE FASTLab™ cassette used to synthesise **[^{18}F]7**

4.0 HPLC chromatograms

Analytical HPLC chromatograms were produced using an Agilent 1200 series or a Shimadzu LC-10Ai Prominence instrument connected to a flow-ram detector (Lablogic, Sheffield, UK).

Variable polarity of the radioactive intermediates in the synthesis of [^{18}F]SqFLT warranted the use two different analytical columns; the same solvent gradient was used for both columns.

Compound **[^{18}F]7** was analysed using a Phenomenex Gemini 5 µm C18 110A, 150 x 4.6 mm column with solvents A) H_2O and B) CH_3CN. Compounds **[^{18}F]9**, **[^{18}F]10** and **[^{18}F]SqFLT** were analysed using a Phenomenex Luna 5 µm Phenyl-Hexyl, 150 x 4.6 mm with solvents A) 0.1% TFA in H_2O and B) CH_3CN.

The gradient is described below:

Time	Flow mL/min	A (%)	B (%)
Initial	1	95	5
1m	1	95	5
16m	1	5	95
18m	1	95	5
20m	1	95	5

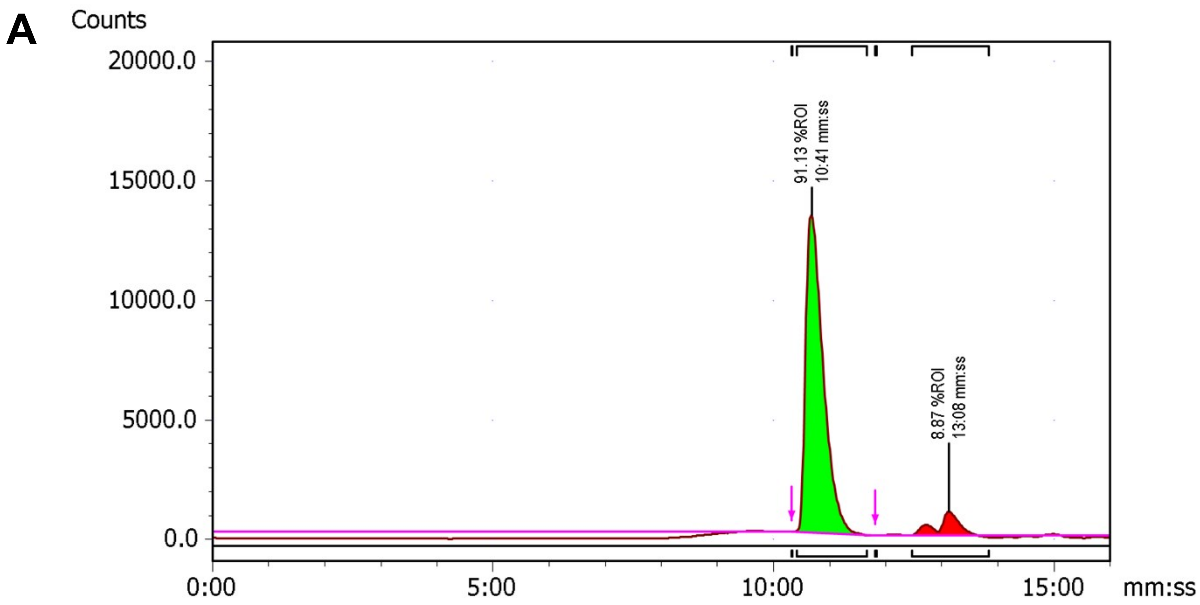

Figure 28. Representative radio-HPLC chromatogram of **[^{18}F]7** (t_R = 10:41 mm:ss) after purification by HLB-SPE.

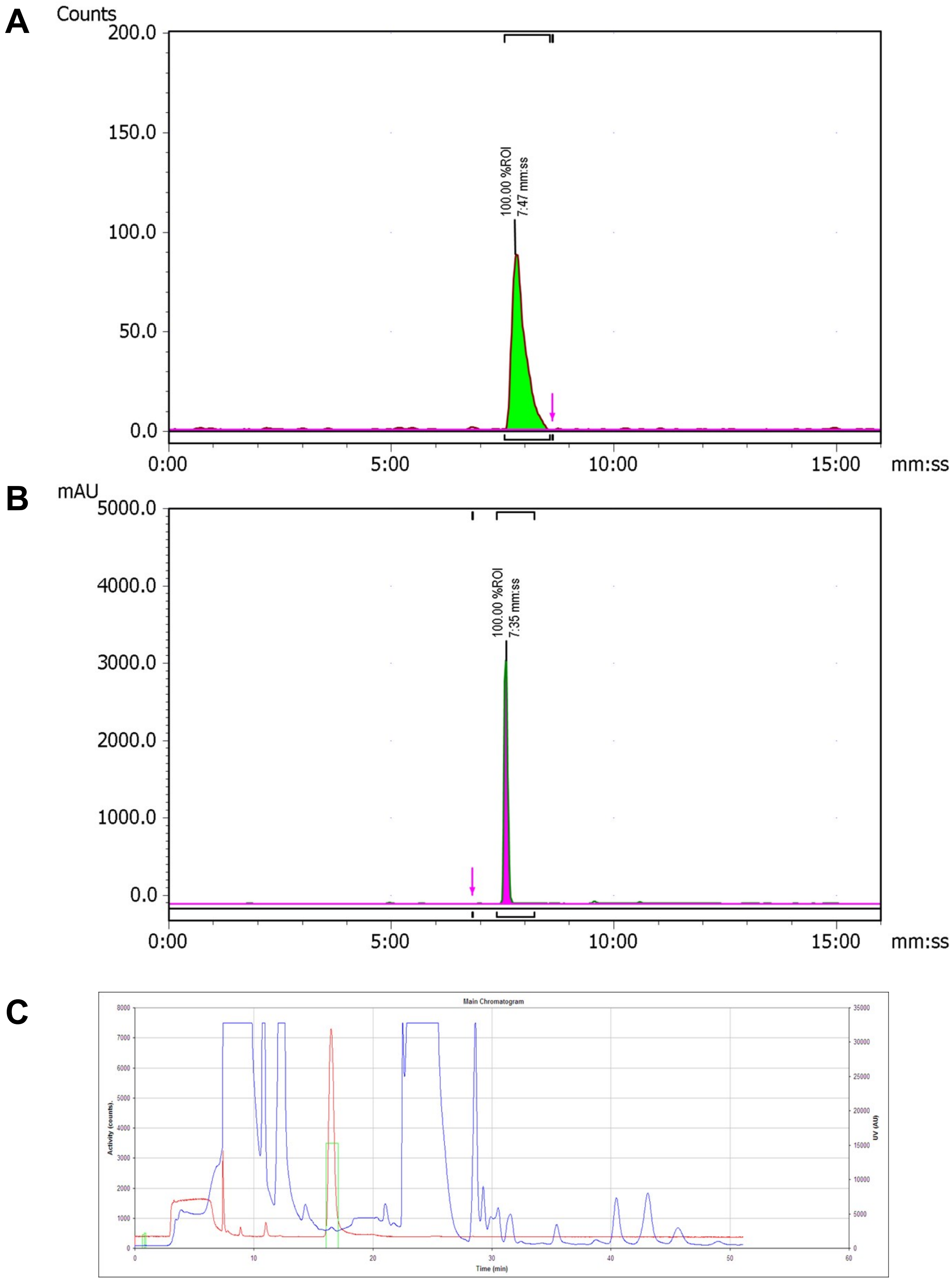

Figure 29. Representative HPLC chromatogram of **[^{18}F]10:** A) radio-HPLC (t_R = 7:47 mm:ss); B) UV-HPLC (254 nm) spiked with reference standard **10**. C) Representative preparative-HPLC chromatogram showing the purification of **[^{18}F]10** (t_R = ca 16 min) Red: radioactive counts; Blue: UV_{254}.

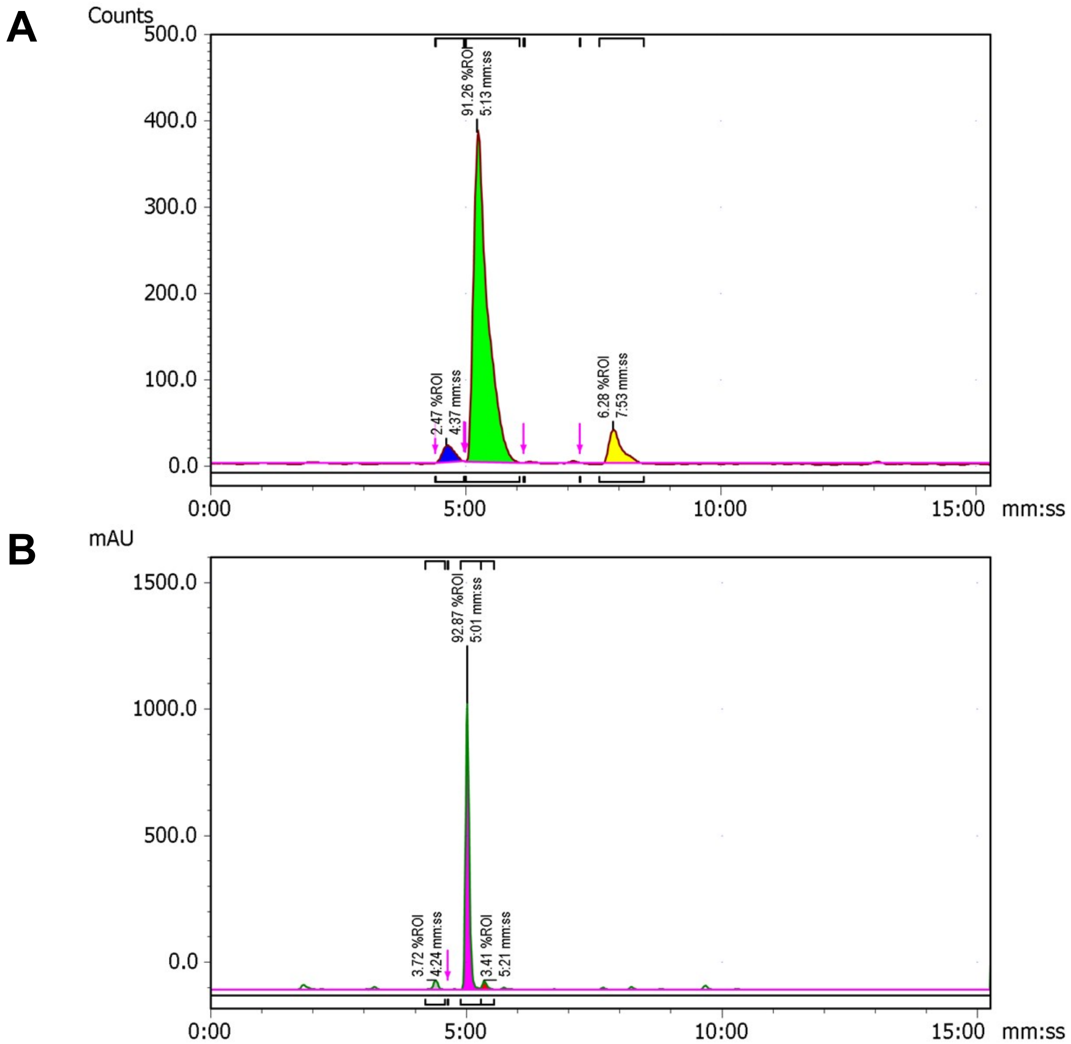

Figure 30. Representative HPLC chromatograms of [^{18}F]SqFLT (t_R = 5:13 mm:ss) **A)** radio-HPLC; **B)** UV-HPLC (254 nm) with spiked [^{19}F]SqFLT reference standard.

5.0 LogD$_{7.5}$ determination

Table 1. Calculated LogP (cLogP) values and measured LogD$_{7.5}$ for [^{18}F]FLT, [^{18}F]FLTMP and [^{18}F]SqFLT.

	Partition coefficient		
**Method**	**[^{18}F]FLT**	**[^{18}F]FLTMP**	**[^{18}F]SqFLT**
LogD$_{7.5}$	ND	ND	-2.90 ± 0.24[b]
cLogP	-0.74	-1.15	-1.20

ND = not determined.
[a] Calculated using Chemdraw 16.0 (Cambridgesoft, USA)
[b] Performed n = 3 with triplicate measurements, represented as Mean ± SD.

6.0 Metabolite analysis in microsomes

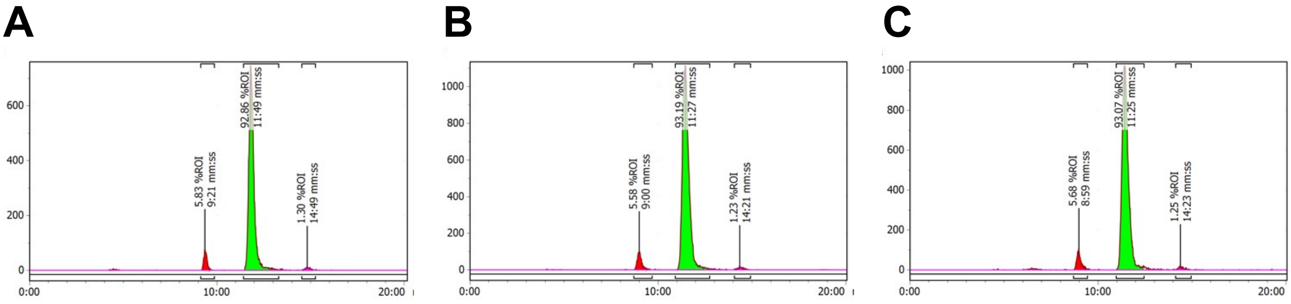

Figure 31. Representative HPLC chromatograms showing **A)** parent [^{18}F]SqFLT, and incubation of [^{18}F]SqFLT with HLM for **B)** 30 mins and **C)** 60 mins. The extraction efficiency was 96.1 ± 0.4 %. The experiment was performed in triplicate (n = 3)

7.0 Gene structure and positions of CRISPR and PCR primers.

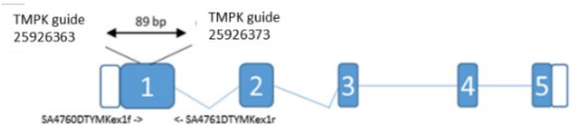

Figure 32. Diagram (not to scale) indicates overall gene structure and positions of the CRISPRS and PCR primers. DNA sequence heterogeneity resulting from Cas9 targeting by CRISPR 25926363 – 25926373.

8.0 Determining if [^{18}F]SqFLT is a substrate for TK1 and ENT1/2

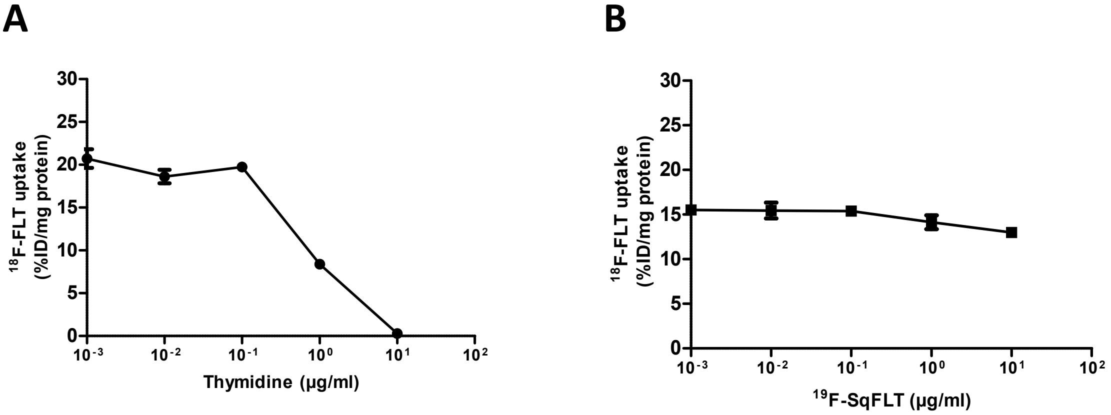

Figure 33. Competition assay using pharmacological doses (10^{-3} - 10^{1} µg) of **A**) thymidine and **B**) [^{19}F]SqFLT to determine the effect on [^{18}F]FLT uptake. HCT116 cells (at 60 – 70 % confluence) were incubated with either thymidine or [^{19}F]SqFLT at multiple concentrations for 20 min prior to co-incubation with [^{18}F]FLT (0.74 MBq). Radioactive uptake normalised to total protein was plotted against log thymidine/[^{19}F]SqFLT concentrations. (n = 5, Mean ± SEM)

9.0 Determining if [^{18}F]SqFLT is a substrate for multidrug resistance proteins

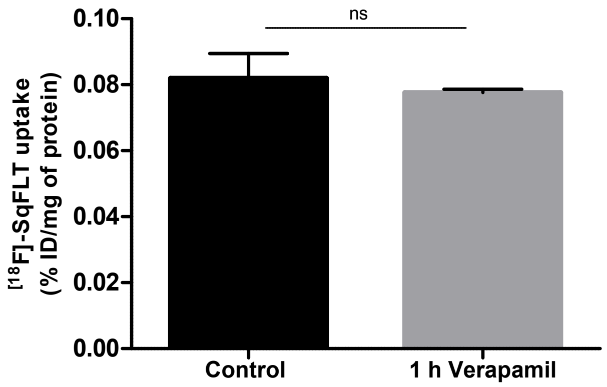

Figure 34. Uptake of [^{18}F]SqFLT in HCT116 cells following treatment with Verapamil. HCT 116 cells were pre-incubated with Verapamil (3 µM) for 60 min followed by co-incubated with [^{18}F]SqFLT (0.74 MBq). One-way Anova statistical analysis and Dunnets post hoc was performed for n = 5 samples.

10.0 Time activity curves (TAC)

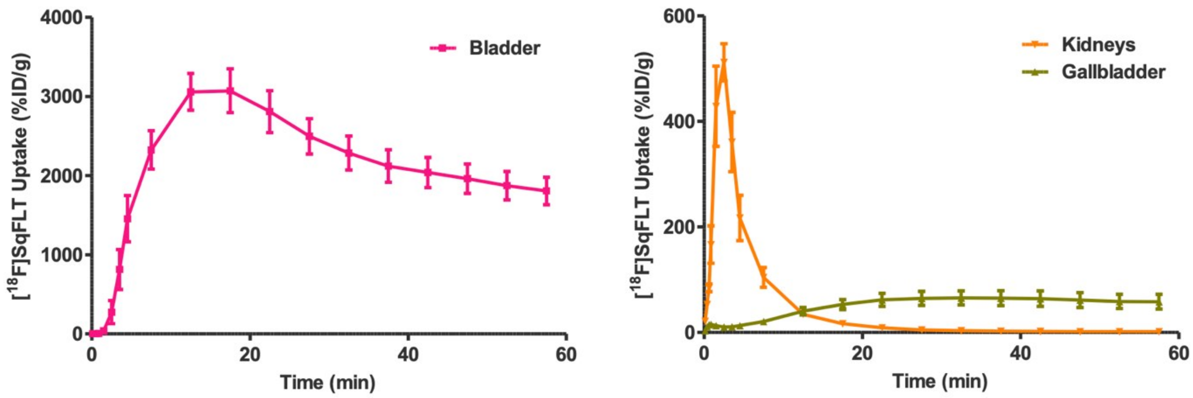

Figure 35. Time-activity curves derived from region-of-interest analysis of PET imaging data (including detail of uptake kinetics between 0-5 min of [^{18}F]SqFLT injection) in bladder, kidneys and gallbladder. Data represent mean ± SEM (n>5) and is expressed as % of injected dose per gram (%ID/g).

11.0 References

1 M. Yun, S. J. Oh, H. J. Ha, J. S. Ryu and D. H. Moon, *Nucl. Med. Biol.*, 2003, **30**, 151–157.

2 C. N. Tetzlaff, I. Schwope, C. F. Bleczinski, J. A. Steinberg and C. Richert, *Tetrahedron Lett.*, 1998, **39**, 4215–4218.
